# Global Transcription Profiles of *Anaplasma phagocytophilum* at Key Stages of Infection in Tick and Human Cell Lines and Granulocytes

**DOI:** 10.3389/fvets.2020.00111

**Published:** 2020-03-06

**Authors:** Curtis M. Nelson, Michael J. Herron, Xin-Ru Wang, Gerald D. Baldridge, Jonathan D. Oliver, Ulrike G. Munderloh

**Affiliations:** ^1^Department of Entomology, College of Food, Agriculture, and Natural Resource Sciences, University of Minnesota, Minneapolis, MN, United States; ^2^Division of Environmental Health Sciences, School of Public Health, University of Minnesota, Minneapolis, MN, United States

**Keywords:** human anaplasmosis, tick-borne pathogen, tiling microarray, obligate intracellular bacterium, *Ixodes scapularis*, differential gene expression, host-cell invasion, intracellular replication

## Abstract

The incidence of human diseases caused by tick-borne pathogens is increasing but little is known about the molecular interactions between the agents and their vectors and hosts. *Anaplasma phagocytophilum* (Ap) is an obligate intracellular, tick-borne bacterium that causes granulocytic anaplasmosis in humans, dogs, sheep, and horses. In mammals, neutrophil granulocytes are a primary target of infection, and in ticks, Ap has been found in gut and salivary gland cells. To identify bacterial genes that enable Ap to invade and proliferate in human and tick cells, labeled mRNA from Ap bound to or replicating within human and tick cells (lines HL-60 and ISE6), and replicating in primary human granulocytes *ex vivo*, was hybridized to a custom tiling microarray containing probes representing the entire Ap genome. Probe signal values plotted over a map of the Ap genome revealed antisense transcripts and unannotated genes. Comparisons of transcript levels from each annotated gene between test conditions (e.g., Ap replicating in HL-60 vs. ISE6) identified those that were differentially transcribed, thereby highlighting genes associated with each condition. Bacteria replicating in HL-60 cells upregulated 122 genes compared to those in ISE6, including numerous *p44* paralogs, five *HGE-14* paralogs, and 32 hypothetical protein genes, of which 47% were predicted to be secreted or localized to the membrane. By comparison, 60% of genes upregulated in ISE6 encoded hypothetical proteins, 60% of which were predicted to be secreted or membrane associated. In granulocytes, Ap upregulated 120 genes compared to HL-60, 33% of them hypothetical and 43% of those predicted to encode secreted or membrane associated proteins. HL-60-grown bacteria binding to HL-60 cells barely responded transcriptionally, while ISE6-grown bacteria binding to ISE6 cells upregulated 48 genes. HL-60-grown bacteria, when incubated with ISE6 cells, upregulated the same genes that were upregulated by ISE6-grown bacteria exposed to uninfected ISE6. Hypothetical genes (constituting about 29% of Ap genes) played a disproportionate role in most infection scenarios, and particular sets of them were consistently upregulated in bacteria binding/entering both ISE6 and HL-60 cells. This suggested that the encoded proteins played central roles in establishing infection in ticks and humans.

## Introduction

*Anaplasma phagocytophilum* (Ap) is an obligate intracellular bacterium that is spread by blacklegged ticks (*Ixodes scapularis* and *Ixodes pacificus*). It causes human anaplasmosis, a serious disease that has steadily increased in prevalence in the United States from 350 cases in 2000 to 5,762 in 2017 (https://www.cdc.gov/anaplasmosis/stats/index.html). Ap alternately infects ticks and mammals to perpetuate itself. In humans and other mammals, infected cells include microvascular endothelium which participates in antigen presentation, and granulocytes that are an important component of the innate immune system. This steers the host toward an ineffective, pro-inflammatory T2-dominated response that induces high fever and liver damage characteristic of human anaplasmosis. A T2 immune response is dominated by production of antibodies as opposed to induction of cytotoxic lymphocytes (a T1 response). The former is ineffective in protecting the host against primary infection with intracellular pathogens (1, 2).

Cell lines from specific host species and tissues are invaluable research tools for analysis of intracellular pathogen–host interactions at the cellular and molecular levels to identify specific host proteins and pathogen factors at play. As models of human infection, relevant cell lines include HMEC-1, a microvascular endothelial cell line, and HL-60, representing human granulocyte precursors, while the tick cell line ISE6 from *Ixodes scapularis* embryos commonly serves to investigate tick infection (3–5). *In vitro* studies further benefit from the availability of complete genome sequences for both hosts and Ap. Despite the many advantages offered by established cell lines for analysis of intracellular bacteria, including ready availability of a well-defined and standardized system, *ex vivo* use of a definitive target cell can reveal molecular mechanisms of pathogenesis that may not otherwise be evident. With the notable exception of granulocytes, cells derived from peripheral blood are conveniently accessible and tractable. Neutrophil granulocytes, however, are first line responders of the innate immune system, and though relatively abundant, when placed *ex vivo*, they rapidly induce defense responses to stimuli designed to efficiently kill invading pathogens. This and their short life span pose special challenges that must be overcome when working with them in a culture system *in vitro* to prevent inadvertent activation and avoid use of senescent cells (6).

Ticks and humans are separated by a large evolutionary distance and are biologically widely divergent, which presents unique challenges to intracellular pathogens that infect these disparate hosts using the limited resources of their small genomes. We have previously shown that Ap activates transcription from specific sets of genes depending on the host cell within which they reside, which is even true for different cell types from the same species (7). These earlier experiments used bacteria harvested from asynchronous, heavily infected cultures, and were not designed to determine a detailed time-course of gene activation during cell invasion and the subsequent intracellular life cycle of Ap, nor did they track changes induced when Ap encounters the alternate host. In the current manuscript, we present a picture of sequential gene activation in bacteria harvested from HL-60 or ISE6 cells and then incubated with fresh uninfected cells. As a control, Ap were held in sterile culture medium in the absence of any host cell stimuli. Samples were collected during phases corresponding to host cell adhesion and invasion, and subsequent intracellular replication. All transcripts, whether from annotated or unannotated genome regions, coding or non-coding DNA strands, were mapped to the genome of the specific Ap isolate used, and quantified. The knowledge gained will facilitate identification of function of the many hypothetical protein genes (those without any known homologs in the data bases) in the Ap genome, as well as provide a basis for rational selection of molecular targets for the prevention or cure of human anaplasmosis.

## Materials and Methods

### Cells, Ap Isolate, and Culture Conditions

HL-60 (human promyeloblast, ATCC #CCL240) cells were maintained between 1 × 10^5^ and 1.5 × 10^6^ cells/mL in RPMI-1640 supplemented with 2 mM glutaMAX, 2 mM L-glutamine, 25 mM HEPES, and 10% FBS (HL-60 medium) in a water saturated, 5% CO_2_ atmosphere at 37°C. ISE6 (*I. scapularis*) cells were maintained as previously described (3) except they were kept at 34°C and the culture medium for Ap-infected cells contained 10% FBS. Human peripheral blood granulocytes were isolated from 5 mL of blood anticoagulated with 50 μL of 0.5 M EDTA from a healthy adult using Ficoll-Paque PREMIUM (GE Healthcare). Blood was mixed with 5 mL of room temperature (RT) RPMI-1640 containing 25 mM HEPES and 2 mM GlutaMAX. Tubes containing 3 mL of Ficoll-Paque were overlaid with 4 mL of diluted blood and centrifuged at 400 × g for 35 min at 20°C in a swinging bucket rotor with the brake off. Layers above the granulocyte/erythrocyte layer were discarded and granulocytes were aspirated (~1.0 mL) from the erythrocyte layer and mixed with 10 mL of RBC Lysis Solution (Qiagen, United States). After 10 min at RT, the erythrocyte-free granulocytes were centrifuged at 400 × g for 10 min at 20°C, washed once with 5 mL of HL-60 medium, and resuspended in 1 mL of medium. HGE1 (passage 10–20), a sequenced [(8), APHH00000000.1] human Ap isolate from Minnesota (9) was used throughout the study. To maintain HGE1 in HL-60, a 4 mL-culture of 10^5^ HL-60 cells/mL was inoculated with 50 μL of HGE1-infected HL-60 (50–90% of infected cells) every 3–4 days. To maintain HGE1 in ISE6, a light monolayer of ISE6 (~3 × 10^6^ cells) in a 25-cm^2^ flask containing 5 mL of medium for Ap-infected cells was inoculated with 100 μL of ~90% infected ISE6 cells. The sealed flask was incubated at 34°C and fed three times per week until the cells began to lift off at about 2 weeks, when the culture was again ~90% infected.

### Cell-Free Bacteria Preparation

Routine maintenance procedures as described above result in asynchronous infections, in which only a proportion of bacteria represent the infectious, dense-core form (10). To achieve standardized infections in HL-60 and ISE6 cultures that produced maximum numbers of infectious bacteria needed for experiments, we used saturating levels of cell-free bacteria at a multiplicity of infection (MOI) of 10–50 bacteria/cell (11) for two successive passages. This resulted in synchronously infected samples for tiling array analysis. To harvest cell-free bacteria from such standardized HL-60 cultures, 3–5 × 10^6^ cells that were ≥95% infected (~72 h post-inoculation, pi, determined by microscopic examination of Giemsa-stained cells deposited onto slides using a Cytospin centrifuge; Thermo Scientific) were resuspended in 3 mL of culture medium. The cell suspension was passed six times through a syringe fitted with a 27-gauge needle to release bacteria, intact cells were removed by centrifugation (710 × g, 6 min), and the supernatant was passed through a 2 μm syringe filter to remove cell nuclei and debris. Bacteria were collected from the filtrate (5,000 × g for 5 min), and resuspended in 100 μL of culture medium. These HL-60-derived bacteria were used either for propagation (another round of HL-60 infection) or to produce samples of infected HL-60 cells or human granulocytes for array analysis. To prepare cell-free bacteria from tick cells, a culture of 3–5 × 10^6^ infected ISE6 cells (about 14 days pi) was resuspended in 3 mL of culture medium, and bacteria were isolated as described above.

### Sample Preparation

The experimental conditions that Ap bacteria were subject to and host cells of origin are listed in Table 1, and are subsequently referred to by their assigned numbers (samples 1–8).

**Table 1:** Samples for RNA extraction.

**Sample Nr./Replicates^a^**	**Origin host cell^b^**	**Target host cell^c^**	**Control^d^**	**Sampling time^e^**
1/1	Ap from HL-60	HL-60	Ap w/o host cells	2 h
2/3	Ap from HL-60	HL-60	Ap w/o host cells	4 h
3/1	Ap from ISE6	ISE6	Ap w/o host cells	2 h
4/3	Ap from ISE6	ISE6	Ap w/o host cells	4 h
5/1	Ap from HL-60	ISE6	Ap w/o host cells	2 h
6/4	Ap from HL-60	HL-60	Uninfected HL-60	24 h
7/3	Ap from ISE6	ISE6	Uninfected ISE6	48 h
8/3	Ap from HL-60	PMN^f^	–	24 h

a Sample number and number of biological replicates used.

b *Host cells from which A. phagocytophilum were purified*.

c *Host cells to which purified A. phagocytophilum were exposed*.

d *Purified A. phagocytophilum incubated in freshly-made cell culture medium alone*.

e *Time post of addition of A. phagocytophilum to target host cells when samples were taken*.

f *Human granulocytes*.

Transcript signals from the entire chromosome of Ap isolate HGE1 (representing the positive and the negative strand, including all coding, non-coding, annotated, and unannotated regions) were measured at two time-points when bacteria were binding to and entering HL-60 or ISE6 cells (2 and 4 h, respectively; Tables S3, S4), and at one time-point when bacteria were replicating in each cell type: 24 h for HL-60 and human granulocytes, and 48 h for ISE6 (Tables S1, S2, S8). All replicates were biological replicates.

To test gene expression in bacteria derived from HL-60 cells and exposed to or cultured in fresh HL-60 cells or human granulocyte samples (samples 1, 2, 6, and 8), 2 × 10^6^ cells in 0.5 mL of HL-60 medium were mixed with 30 μL of cell-free bacteria (MOI: 10–50) in 1.5 mL microfuge tubes and incubated at 37°C for 2 h (sample 1) or 4 h (sample 2). Tubes were manually inverted to gently mix contents every 15 min to maximize contact between bacteria and cells. Cells were allowed to settle for a final 15 min period, and the medium containing unbound bacteria was aspirated. Cells with bound bacteria were washed once by centrifugation at 350 × g for 6 min in 5 mL of medium pre-warmed to 37°C, and dissolved in 1 mL of Tri Reagent (Sigma-Aldrich) to extract RNA for measurement of transcription associated with cell binding/entry. To measure transcription when bacteria were replicating, cells infected as described were resuspended in 20 mL of medium and further incubated at 37°C for 24 h (samples 6 and 8). They were then pelleted (350 × g, 6 min) and dissolved in 1 mL of Tri Reagent. Control samples consisted of 30 μL of cell-free bacteria prepared identically in parallel in 0.5 mL of medium alone, to measure bacterial transcription after incubation in the absence of host cells, or 2 × 10^6^ HL-60 cells or 3 × 10^6^ ISE6 cells without Ap (samples 6 and 10, respectively). Following collection by centrifugation at 4,000 × g for 6 min, bacteria were dissolved in 1 mL of Tri Reagent. Granulocytes are less transcriptionally active than other leukocytes or HL-60 cells (12), and thus contribute much less cellular mRNA. To equalize the RNA concentration in sample 8 to the other samples, 1 × 10^6^ sterile HL-60 cells were added to sample 8 after addition of Tri Reagent. The same was done with control samples of bacteria in fresh medium alone, as described below under RNA isolation. For ISE6 samples, 3 × 10^6^ ISE6 cells established as a light monolayer (~80% confluent) in a 25-cm^2^ flask were inoculated with 30 μL of ISE6-derived bacteria in 1 mL of ISE6 growth medium (MOI: 10–50) and incubated for 2 h (sample 3) or 4 h (sample 4) at 34°C with gentle hand rocking at 15-min intervals to redistribute the bacteria across the monolayer. To remove unbound bacteria, the medium was discarded, and the monolayer rinsed with 5 mL of medium warmed to 34°C, then flooded with 3 mL of Tri Reagent to collect RNA from bacteria binding/entering ISE6. To measure gene transcription when bacteria were replicating in ISE6 cells, cultures were incubated for another 48 h at 34°C in 5 mL of medium (sample 7), and RNA was then isolated from the monolayer as above.

**Figure 1:**
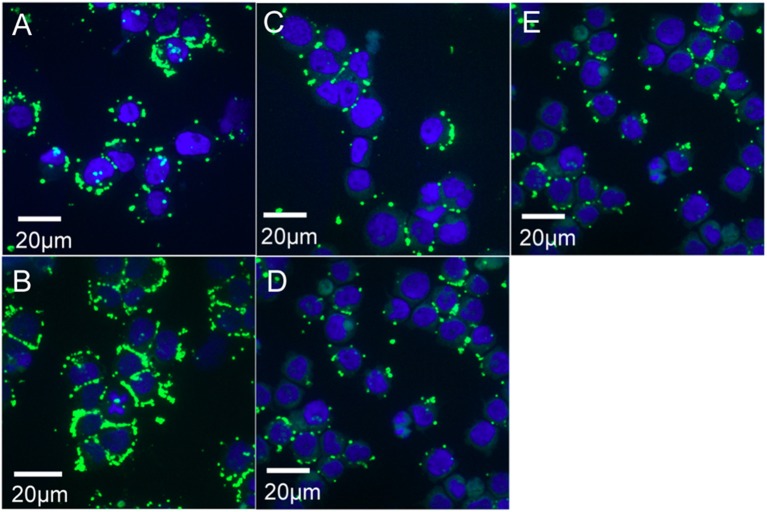
Confocal microscopy images of *A. phagocytophilum* (Ap) binding/entering HL-60 and ISE6 cells. Cell nuclei are stained blue with DAPI, bacteria green by immunofluorescence (primary antibody: dog anti-Ap serum; secondary antibody: goat anti-dog IgG-FITC). **(A)** Sample 1 (Ap incubated with HL-60 for 2 h; **(B)** Sample 2 (Ap incubated with HL-60 for 4 h); **(C)** Sample 3 (Ap incubated with ISE6 for 2 h; **(D)** Sample 4 (Ap incubated with ISE6 for 4 h); and **(E)** Sample 5 (HL-60-grown bacteria incubated with ISE6 cells for 2 h).

To visualize Ap binding/entering HL-60 cells at 2 and 4 h, 1 × 10^4^ cells from preparations used for RNA isolation (samples 1 and 2; Figures 1A,B), were deposited onto microscope slides using a Cytospin centrifuge, air dried five min, and fixed in methanol five min. Bacteria were labeled with antibody by incubating first with dog anti-Ap serum (1/2,000 dilution) followed with goat anti-dog IgG-FITC (13). Preparations were overlaid with PBS containing 0.15 μg DAPI/mL to stain HL-60 cell nuclei (blue). For Ap exposed to ISE6 for 2 and 4 h (corresponding to samples 3, 4, and 5; Figures 1C–E), cells were first established in glass bottom dishes (MatTek Corporation) then incubated with bacteria at the same concentration and MOI as for microarray samples. After washing, cells and bacteria were fixed by flooding dishes with methanol, air dried, stained as above, and imaged within the dishes. Cells with bound Ap were viewed on an Olympus BX61 confocal microscope (Olympus America, Pennsylvania) equipped with a DSU-D2 confocal disk-scanning unit, and images were acquired with a Photometrics QuantEM:512SC EMCCD camera (Photometrics, Arizona) or a QFire color camera (Qimaging, California).

### RNA Isolation

Total RNA for each sample was extracted from 2 × 10^6^ infected HL-60 cells or 3 × 10^6^ infected ISE6 cells, and purified according to the product protocol. Finally, the RNA pellets were washed in 75% ethanol and stored in fresh 75% ethanol at −20°C for 1–2 weeks. For array analysis, ethanol was removed, the RNA pellet was dried briefly, dissolved in 30 μL RNase-free water, and sent on dry ice to MOgene, LC (St. Louis, MO) where 2 μg of each sample was used for labeling, array hybridization, and scanning (14). The RNA content of control samples of bacteria incubated in fresh medium alone was adjusted to that in samples of infected cells by adding an equal number of the appropriate sterile host cells (2 × 10^6^ HL-60 or 3 × 10^6^ ISE6) to the control samples immediately after the bacteria had been dissolved in Tri Reagent.

### Tiling Microarray Design

The Agilent microarray design tool, eArray, was used to design a custom, whole genome tiling array for HGE1 based on its genomic sequence (contig 1 GI: 546157146, contig 2 GI: 482614209; accessible at NCBI, numbers APHH01000001.1 and APHH01000002.1, respectively) and was manufactured by Agilent in an 8-plex format (eight arrays per slide). Each array contained 60,000 60-mer oligonucleotides, each overlapping its genomic neighbor by 10 bases to represent all genomic sequences on both DNA strands, allowing detection and relative quantification of any transcribed RNA, whether from annotated genes, intergenic regions, or sequences on the opposite (non-coding) DNA strand (antisense transcripts).

### Microarray Analysis

In our first approach to preparing labeled samples (i.e., targets) from Ap culture RNA, Agilent's FairPlay III kit was used according to manufacturer's instructions. This kit employs a mutant of the MMLV reverse transcriptase and random primers to generate cDNA labeled with Cy3. To prevent second strand cDNA synthesis, actinomycin D (6 μg/mL) was incorporated into the reverse transcription step (15). In our second approach, which was used to generate all data presented here, total RNA isolated from Ap-infected host cells was directly labeled chemically using the Kreatech (Leica Biosystems) ULS Labeling Kit and protocol. A platinum complex linked to Cy3 forms a coordinate bond with guanine at the N7 position of RNA, and no enzyme is involved. With this method, 2 μg of labeled RNA is sufficient for array hybridization, which serves to reduce background noise (14). Labeled RNA was fragmented with 1X Agilent fragmentation buffer at 60°C for 30 min, and the reaction was quenched by the addition of hybridization buffer. Samples were hybridized with the tiling arrays at 65°C for 17 h with continuous mixing at 10 rpm and scanned on an Agilent C high-resolution scanner with 20-bit imaging.

### Array Data Analysis

**Figure 2:**
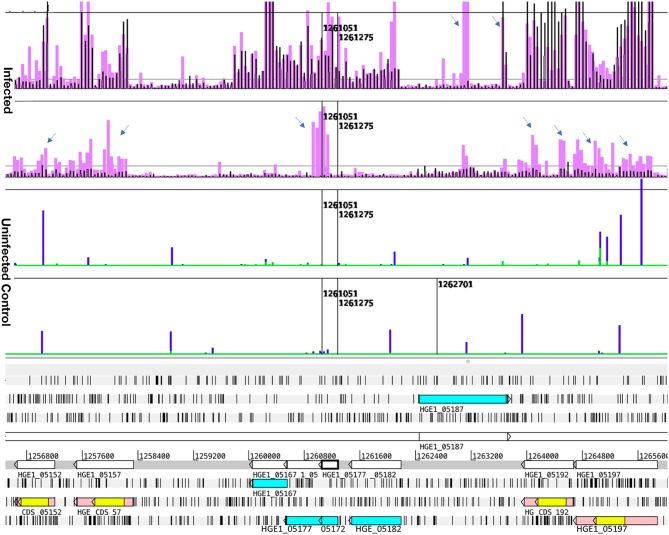
Transcript levels in Sample 1 and sample 3, illustrating differential *p44* transcription in HL-60 and ISE6, and antisense transcripts. Transcription data (top four graphs) plotted over the annotated genome sequence (sections at the bottom; turquoise boxes denote coding regions of annotated genes, pink boxes denote *p44* conserved sequences with yellow boxes denoting their center hypervariable region) of *A. phagocytophilum* (Ap) strain HGE1 using the Artemis genome browser. Black bars represent *A. phagocytophilum* (Ap) transcription in sample 1 (2 h with HL-60 cells), and lavender bars transcription signal in sample 3 (2 h with ISE6 cells) (Infected). Each bar corresponds to one 60-mer probe on the tiling array. The bottom (Uninfected) graphs depict lack of signal from uninfected controls (HL-60 green, ISE6 blue). Prominent antisense signals in sample 3 are indicated (arrows), especially at the junction of HGE1_05172 (putative ATP synthase FO, B subunit) and HGE1_05177 (ATP synthase subunit C), but also from the *p44* paralogs (pink bars with yellow centers) and HGE1_05187. HGE1_05192 (*p44*-18) has strong signal corresponding to its hypervariable region (yellow) indicating that it is specifically transcribed in sample 1. Nucleotide positions in the genome are indicated by numbers above the gray line.

We extracted probe intensity data from tabular text files provided by MOgene, reformatted them, and intermittent spikes with intensities >5 times the sum of immediately adjacent probes were filtered by replacing them with the average of those values, as done previously (7). Data from independent samples were quantile normalized (16) except where otherwise noted. Transcription values for entire genes (1,189 locus tags) were calculated as the sum of hybridization values of all probes corresponding to each gene. The 1,189 locus tags measured included 13 truncated *p44* pseudogenes consisting of only conserved sequence, referred to as *p44* fragments, and 93 others that contained conserved and hypervariable sequence (HVR). Since actually transcribed pseudogenes should show elevated HVR signals, all 93 pseudogenes were visually assessed in Artemis plots under each of the 12 conditions tested, with signal levels from only the HVRs rated on a scale from 0 (not transcribed) to 4 (strongly transcribed), as listed in Table S9 and illustrated in Figure 2. Gene transcription values from samples with three or more replicates were compared using the paired two-tailed Student's t-test to those in relevant samples (e.g., Ap replicating in HL60 vs. in ISE6). Values that were statistically significant (*p* ≤ 0.05) and ≥1.5- or 2-fold elevated or reduced relative to comparison samples were identified as differentially transcribed and ranked in order of transcript abundance. Differential transcription values from single sample comparisons (2-h incubations) were not assessed for statistical significance and genes were ranked only if differing by ≥2-fold. For easy visualization of transcripts from genes and intergenic and antisense sequences, transcription profiles of selected genome regions were displayed graphically by plotting hybridization values from 60-mer probes above the annotated genome of HGE1 in the genome browser Artemis (17) (Figures S1–S9).

Raw microarray data have been deposited in the Dryad database, available at https://doi.org/10.5061/dryad.18931zcs5.

## Results

### Sample Labeling

**Figure 3:**
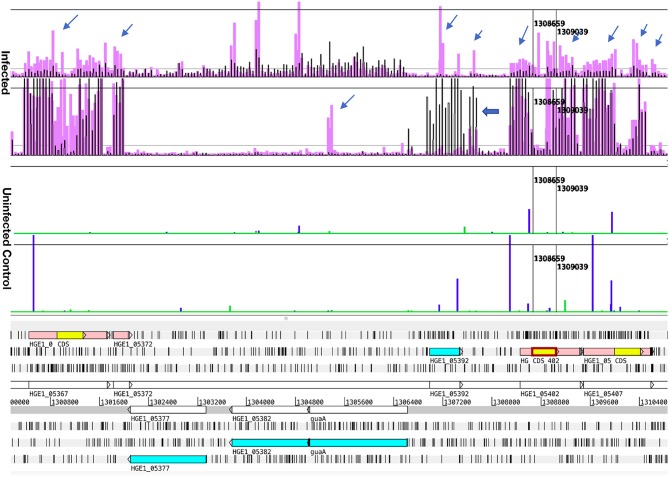
Comparison of transcript signal from Samples 1 and 3, illustrating antisense transcripts and transcription from an unannotated gene. Transcription data (top four graphs) plotted over the annotated genome sequence (sections at the bottom; turquoise boxes denote coding regions of annotated genes, pink boxes denote *p44* conserved sequences with yellow boxes denoting their center hypervariable region) of *A. phagocytophilum* (Ap) strain HGE1 using the Artemis genome browser. The top two graphs (Infected) represent Ap transcription in Sample 1 (black bars, 2 h with HL-60 cells) vs. in Sample 3 (lavender bars, 2 h with ISE6 cells). Each vertical bar corresponds to one 60-mer probe on the tiling array. The two graphs below (Uninfected control) are plots of data from uninfected cells (HL-60, green; ISE6, blue), to demonstrate that host cell transcripts are not responsible for antisense signals. Occasional probes produce signals but overall the sterile cell graphs are flat. Prominent antisense signals generated by Ap from ISE6 are indicated by slender arrows, including those associated with the *p44* conserved regions (pink boxes). Also shown is a *p44* paralog (HGE_05367; at base position 1,300,800 in the genome) specifically transcribed in Sample 3, as indicated by signal corresponding to the center hypervariable region (yellow box). Note a hypothetical gene, HGE1_05392, with elevated transcript levels in Sample 1 (and with antisense signals in Sample 3). Immediately downstream, a transcription peak indicates the presence of a small, unannotated gene (bold arrow). Transcription levels from the HVRs of *p44*s HGE_05367, HGE1_05402, and 05407 were rated 1, 0, and 2 in HL-60, and 4, 0, and 1 in ISE6, respectively. Nucleotide positions in the genome are indicated by numbers above the gray line.

**Figure 4:**
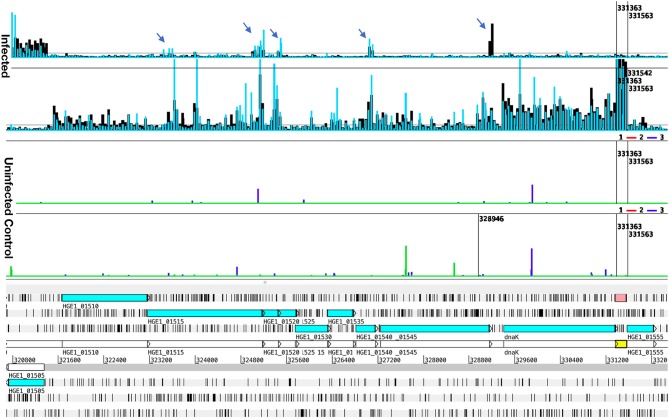
Transcript levels in Samples 6 and 8, illustrating antisense signals and transcription from an unannotated gene. Transcription data (top four graphs) plotted over the annotated genome sequence (sections at the bottom; turquoise boxes denote coding regions of annotated genes) of *A. phagocytophilum* (Ap) strain HGE1 using the Artemis genome browser. Blue and black bars (top two graphs) represent Ap transcription in sample 6 (Ap replicating in HL-60 cells for 24 h, blue bars), and in and sample 8 (Ap replicating in human granulocytes, PMN, for 24 h, black bars). Each bar corresponds to one 60-mer probe on the tiling array. The bottom (Uninfected) graphs depict lack of signal from uninfected controls (HL-60 green, PMN blue). Antisense signals from infected HL-60 cells and granulocytes are indicated (arrows). A strong transcription signal (pink box) is visible from an unannotated gene immediately downstream of dnaK. Nucleotide positions in the genome are indicated by numbers above the gray line.

Addition of actinomycin D during reverse transcription did not prevent synthesis of spurious second strand cDNA when using the FairPlay III kit to generate labeled samples. This was evident when comparing transcription signals from the same sample prepared with either indirectly labeled cDNA or directly labeled RNA (Figure S1). Direct labeling produced stronger sense signals without the prominent false antisense signals seen with the reverse transcriptase-based method. Genuine antisense signals can be seen in Artemis plots of the subsequent transcription data from Kreatech-labeled samples. They were especially common in bacteria infecting ISE6 cells (Figure 3), but can also be seen in infected HL-60 cells and human granulocytes (Figure 4), and were often associated with reduced sense transcription. The conserved sequences of *p44* pseudogenes consistently showed antisense signals. Anti-sense transcripts were not derived from host cell mRNA (Figures 1–3, Uninfected Control).

### Transcription Associated With Host Cell Binding/Entry

Table 2 lists the most differentially regulated genes in the various culture conditions examined.

**Table 2:** Most differentially regulated genes in the cell culture systems used in this study.

**Sample**	**Locus tag**	**Function**	**Ratio (*P*-value)**
Sample 6 (24 h in HL-60) vs. Sample 7 (48 h in ISE6)	HGE1_00140	Hypothetical, localizes to morulae membrane (18)	5.89 (0.045)
	HGE1_05192	*p44-18*	3.83 (0.008)
	HGE1_04712	Phage repressor protein C	3.92 (0.026)
Sample 5 (HL-60 grown Ap exposed to ISE6) vs. Sample 1 (2 h with HL-60)	HGE1_05312	Transcriptional regulator tr1 (at p44 expression locus) (19, 23, 24)	9.94 (not done)
	HGE1_02797	Hypothetical	6.06 (not done)
	HGE1_02157	Single-stranded DNA-binding protein	5.97 (not done)
Sample 6 (24 h in HL-60 vs. 24 h in PMN (sample 8)	HGE1_01702	tRNA-Arg	7.561 (0.018)
	HGE1_06226	tRNA-Lys	7.45 (0.050)
	HGE1_06206	tRNA-Met	5.36 (0.000)

*HL-60, human promyelocyte cells; ISE6, Ixodes scapularis tick cells; PMN, human granulocytes*.

Transcript profiles from sample 1 (Ap from HL-60 cells exposed to uninfected HL-60 cells for 2 h) showed that three genes were ≥2-fold elevated with HL-60 cells compared to six in the fresh medium control (Table S3A). Comparison of gene transcription values from sample 2 (4 h with HL-60) to those of the control samples showed that none were differentially transcribed (≥2-fold elevated, *p* ≤ 0.05). Thus, HL-60-produced bacteria that bound to/entered fresh HL-60 cells showed little transcription induced by cell contact, suggesting that before 2 h, and probably upon release from parent cells, the genes necessary for HL-60 cell binding and entry were already expressed. To determine if there was a change in transcription over time, samples 1 and 2 were compared to each other, identifying seven genes with elevated transcript levels at 2 h (three that encode tRNAs) vs. 45 at 4 h (Table S3B). Because the comparison of sample 2 (4 h with HL-60 cells) to controls showed no differences, upregulation of genes at 4 h compared to 2 h occurred through passage of time rather than as a result of bacterial interactions with HL-60 cells.

Bacteria bound to and entering ISE6 host cells after 2 h incubation (sample 3) showed increased mRNA levels for 48 genes relative to the medium only control in which 24 genes were upregulated (Table S4A). After 4 h with ISE6 (sample 4), eight genes had elevated levels (2.0- to 8.7-fold) vs. 37 in controls (Table S4B). The 48 genes with elevated transcription in sample 3 (2 h incubation with ISE6) included 18 hypothetical genes of which ten were predicted by CELLO or secP to be localized to the bacterial membranes or to be secreted. Additional genes with elevated transcript levels were: the *tr1* transcription regulator (HGE1_05312, 3.4-fold) that was also upregulated in sample 7 (Ap replicating in ISE6) and sample 8 (Ap replicating in granulocytes) relative to sample 6 (Ap replicating in HL-60); HGE1_03902 [2.8-fold; encoding the invasin AipA; (25)]; three *vir* genes (two *virB2* and *virB6*); and an *HGE-14* paralog (HGE1_01777) that was not among those paralogs upregulated during replication in any of the cell types. The 24 genes with elevated mRNA signals in the 2 h control for sample 3 included 10 hypothetical genes. Eight genes upregulated in sample 4 (4 h in the presence of ISE6 cells) included two that encode guanylate kinases (8.7- and 4.5-fold elevated), which have roles in nucleotide transport and metabolism, three hypothetical genes (one predicted by secP to encode a secreted protein), and two with roles in translation, ribosomal structure and biogenesis (Table S4B). The 37 upregulated genes in the controls for sample 4 included 12 *p44* pseudogenes and omp-1A, six hypothetical genes, six that encode tRNAs, and two *virB2* genes. Thus, bacteria incubated for 2 h with ISE6 cells (sample 3) responded to them specifically, as 67% of differentially transcribed genes were upregulated in comparison to the control. At 4 h (sample 4), 82% of genes were upregulated in the control, indicating that most of the host cell-specific response occurred by 2 h.

Comparison of the two ISE6 samples 3 and 4 (2 vs. 4 h; Table S4C) identified 232 differentially transcribed genes; 167 were upregulated in sample 3 (2 h), including 50 *p44* pseudogenes (vs. none in sample 4); 22 tRNA genes (vs. five in sample 4); 14 genes with roles in translation, ribosomal structure and biogenesis (vs. three in sample 4); ten genes involved in post-translational modification, protein turnover, chaperones (vs. three in sample 4); seven genes involved in intracellular trafficking, secretion, and vesicular transport (vs. one in sample 4), and six genes with roles in replication, recombination and repair (vs. one in sample 4) (Table S4C). In sample 3, *p44* pseudogenes along with recombination- and secretion-related genes were upregulated compared to sample 4, in which the gene encoding major surface protein 4 (*msp4*) was upregulated and hypothetical genes predominated. Of these, 57% were predicted by secP, sigP, or CELLO to be secreted or localized to the bacterial membranes or periplasm, including HGE1_05392 (12.5-fold upregulated; Figure 1), the product of which is associated with dense core organisms (10), and HGE1_03697 that encodes Ats-1, which is translocated to mitochondria for inhibition of apoptosis, and induction of autophagosome formation (26). Transcription of *p44* was upregulated in sample 3 (Figure 2) and declined in sample 4, but levels remained elevated in the 4 h controls (no host cells), indicating that in addition to causing the upregulation of the genes noted above, bacterial contact with uninfected ISE6 cells was responsible for the drop in *p44* mRNA levels.

Sample 5 (bacteria from HL-60 cells incubated with ISE6 cells for 2 h) was included to determine how bacteria produced in human cells would adjust their transcription pattern to express genes required for tick cell binding and entry. Compared to sample 1 (Ap from HL-60 incubated with fresh HL-60 cells for 2 h), 127 genes were differentially transcribed: 54 in sample 5 (20 hypothetical genes) and 72 in sample 1 (13 hypothetical) (Table S5A). Seventeen of the 21 hypothetical genes with elevated transcripts in sample 5 were also upregulated in sample 3 when compared to sample 1 (below), and eight of these were predicted to encode proteins localized to the bacterial membranes, periplasm, or to be secreted. In addition, the transcription regulator *tr1* (HGE1_05312), shown to be upregulated 9.1-fold in sample 3 (2 h with ISE6) relative to sample 1 (2 h with HL-60; Table S6A), was the most upregulated gene in sample 5 (9.9-fold). Also upregulated in sample 5, and in sample 3 relative to sample 1, was HGE1_00935, which encodes an effector protein that interacts with the trans-Golgi network (27). Through contact with ISE6 cells, the HL-60-grown bacteria (sample 5) shifted their transcription, and early ISE6-specific effectors and membrane proteins were upregulated. When confronted with the homologous host (sample 1), 31 of the 72 genes with elevated transcript levels had roles in translation, ribosomal structure and biogenesis, indicating that transcription had already shifted to pathways that support bacterial growth and replication (Table S5B).

These comparisons showed that the 2 h samples identified host cell-specific transcription, whereas the 4 h samples included time-dependent changes in transcription in addition to those seen at 2 h. Therefore, in subsequent comparisons to identify genes specific to human- and tick-cell binding and entry, and to distinguish replication-specific genes, the 2 h data were used.

### Transcriptional Differences Between Bacteria Binding/Entering HL-60 and ISE6 Cells

To distinguish the earliest human- and tick-cell-specific gene activity, sample 1 (2 h with HL-60) and sample 3 (2 h with ISE6) samples were compared, revealing 205 differentially transcribed (≥2-fold elevated transcripts) genes (Table S6A): 126 in Ap incubated with HL-60, of which 36 were hypothetical genes, and 79 in Ap incubated with ISE6, including 39 hypothetical genes (Figure S4). Of the 36 hypothetical genes with elevated transcript levels in sample 1, 25 encode proteins predicted by secP or CELLO to be secreted or localized to the bacterial membranes or periplasm. Among hypothetical genes, HGE1_03552 was strongly upregulated in sample 3, but not in sample 1, and HGE1_03512, was upregulated at 2 h in sample 1, but not in sample 3. Antisense signals were evident especially in sample 3 from several of the genes, as well as an unannotated peak just upstream of HGE1_03517 (Figure S4). Also upregulated in sample 1 were 25 genes with roles in translation, ribosomal structure, and biogenesis (vs. only three in sample 3); six *HGE-14* paralogs (HGE1_01782, 01772, 01752, 02095, 02107, and 02100, vs. one, HGE1_02092, in sample 3), including three known type IV secretion system (T4SS) substrates translocated to host cell nuclei (21); and five genes each involved in signal transduction mechanisms and energy production and conversion (vs. none in either category in sample 3; Table S6A). Additional notable HGE1 genes with elevated transcripts in sample 1 included: HGE1_05392 (Figure 1) known to be expressed by dense-cored, infectious bacteria [(10), which was 17-fold upregulated; this gene was also upregulated in sample 4]. By contrast, Ap entering ISE6 tick cells did not express HGE1_05392, and in fact, produced strong anti-sense signal possibly indicative of a regulatory mechanism. This result indicates that this gene product does not participate in invasion of ISE6 cells. Similarly, prominent antisense signals in sample 3 are evident at the junction of HGE1_05172 (putative ATP synthase FO, B subunit) and 05177 (ATP synthase subunit C; Figure 2), suggesting this ATP synthase is not required for invasion of ISE6 cells. HGE1_00140, which was 7-fold elevated, had previously been shown to localize to the cytosolic side of the morula membrane (18); *dnaK*, 5-fold elevated, involved in cell infection (28); HGE1_02357 encoding ApxR, 4.8-fold elevated, associated with upregulation of *p44* expression in HL-60 cells (23, 24) but which was not elevated in sample 6 (24 h in HL-60; Table S1A); HGE1_03232, 3.9-fold elevated, encoding the effector AnkA (29), and *omp1A* (HGE1_01505), 3.5-fold elevated, an outer-membrane-expressed protein that interacts with mammalian host cells (30). Genes that were upregulated 2- to 3-fold in sample 1 included: HGE1_03697, encoding Ats-1, which is imported to mitochondria, inhibits apoptosis and induces autophagosome formation (26); HGE1_01090, encoding Asp14, which is surface-expressed and critical for infection (31); and HGE1_03902, encoding AipA, an invasin necessary for infection of mammalian cells (25). HGE1 genes upregulated in sample 3 (2 h with ISE6 cells) included the *p44*ES transcription regulator, *tr1* [HGE1_05312; (19)], which was the second-most elevated (9.1-fold, Figure S5), and likely drives expression of *p44* paralogs specifically expressed in ISE6 cells, i.e., HGE1_05312 (Figure S5), and several others at later times; HGE1_00935 (upregulated 9.9-fold in sample 5) encoding an effector that interacts with the trans-Golgi network (27); and 39 hypothetical genes, 19 of which were predicted to be secreted or localized to the bacterial membranes or periplasm.

### Transcriptional Differences Between Bacteria Binding/Entering and Replicating in HL-60 and ISE6 Cells

For each cell line, the Ap replication/growth samples were compared to the earliest binding/entry sample to further differentiate genes involved in those two infection stages in human and tick host cells. Comparison of sample 6 (24 h in HL-60) to sample 1 (2 h with HL-60) revealed 80 differentially transcribed (≥2-fold elevated) genes, 34 at 24 h and 46 at 2 h. Genes in the 24 h list included 15 that encode hypothetical proteins, six that encode proteins predicted by secP or CELLO to be secreted or localized to the bacterial membranes or periplasm, eight *p44* pseudogenes, and the transcription regulator *tr1* (2.3-fold) (Table S7).

In sample 1 (2 h with HL-60), half of the upregulated genes (23) encoded hypothetical proteins (Table S3A), 18 of which were also upregulated in sample 1 compared to sample 3 (Table S6A); 15 of these encode proteins predicted to be secreted or localized to the bacterial membrane or periplasm. Also upregulated in sample 1 were five *HGE-14* paralogs (Table S3A) that were still upregulated in sample 6 compared to sample 7 (48 h in ISE6; Table S1A), and in sample 1 when compared to sample 3 (Table S6A). In addition, several genes whose products are membrane-expressed or secreted early by infectious bacteria, especially those incubated with HL-60 cells, were upregulated in sample 1 (Table S7).

Comparison of sample 7 (48 h in ISE6) to sample 3 (2 h in ISE6) identified 65 differentially transcribed (≥2-fold elevated) genes, 43 with elevated transcript levels at 48 h and 22 at 2 h (Table S8A). Of those upregulated at 48 h, 20 encode hypothetical proteins, 12 of which were predicted to localize to the bacterial membranes or periplasm or to be secreted. Also upregulated in sample 7 was HGE1_05392, which was previously found to be expressed by infectious bacteria (10). In the present study, it was upregulated 4.4-fold in sample 7 relative to sample 3, but even more so (upregulated 12.5-fold) in sample 4 compared to sample 3 (Table S4C). HGE1_01872, predicted by both secP and sigP to encode a secreted protein, was upregulated in sample 4 (4 h) compared to sample 3 (2 h; Figure S9). Interestingly, *msp4* was 3.6-fold elevated in sample 7, and similarly elevated in sample 4 compared to sample 3 (Table S4C) suggesting a role following invasion. In addition, eight genes involved in translation, ribosomal structure and biogenesis were elevated at 48 h in sample 7 (vs. one in sample 3), which is consistent with the activities of growing/replicating organisms.

In sample 3 (2 h), 11 of 22 upregulated genes encoded hypothetical proteins, five of which were also upregulated when compared to sample 1 (2 h with HL-60; Table S6A). These included HGE1_05647 and HGE1_02122 that were among the 17 upregulated in HL-60-grown bacteria after 2 h incubation with ISE6 cells (sample 5; Table S5A). Another of these early ISE6 hypothetical genes, HGE1_03797, whose product was predicted by secP to be secreted, was the most upregulated gene (27-fold) at 48 h, and was also upregulated at 4 h compared to 2 h (Table S4C), indicating that its role extends beyond the early stages of ISE6 cell infection. Two others on the early ISE6 genes list, HGE1_03547 and HGE1_03167, and one not on the list but upregulated at 2 h with ISE6 vs. 2 h with HL-60, HGE1_03492, were also upregulated at 48 h, indicating that they, too, encode proteins with functions early and at 48 h in ISE6. HGE1_04797, the most upregulated gene at 2 h (relative to 48 h), was upregulated 3.2-fold after 2 h incubation with ISE6 compared to control bacteria (Table S4A), suggesting that its role in ISE6 infection is specifically early. Also elevated at 2 h were three full-length *p44* pseudogenes (Figure S9). In Ap incubated with ISE6 cells, *p44* transcripts were elevated at 2 h, diminished by 4 h, and remained low at 48 h.

### Transcriptional Differences Between Bacteria Replicating in HL-60, Granulocytes, and ISE6

**Figure 5:**
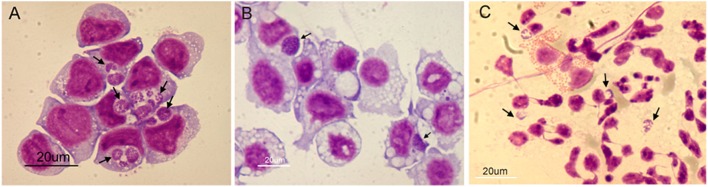
Light microscopy images of Giemsa-stained cells used for transcriptome analysis of *A. phagocytophilum* (Ap) replicating in three cell types. Panel **(A)** HL-60 cells infected 24 h, **(B)** ISE6 cells infected 48 h, and **(C)** human granulocytes infected 24 h. Arrows indicate morulae (Ap colonies within vacuoles). Cytospin slides of each sample were air dried, fixed in methanol and Giemsa-stained. Scale bars = 20 μm.

Figure 5 demonstrates the appearance of infected cells in Giemsa-stained samples to show that morulae containing large numbers of Ap were produced in HL-60 cells (panel A), ISE6 cells (panel B), and human granulocytes (panel C). Transcript levels of all genes from bacteria replicating in the human cell line (sample 6) and human granulocytes (sample 8) vs. those from bacteria replicating in the tick cell line (sample 7) showed host-cell specific profiles when using Ap incubated in culture medium in the absence of the respective host cells as controls. Of 164 differentially transcribed genes (≥1.5-fold elevated, *p* ≤ 0.05), 122 had elevated mRNA levels in HL-60 cells and 42 in ISE6 cells (Table S1A). The HL-60 list was dominated by genes found only in the family Anaplasmataceae (43, 35% of total), hereafter referred to as unique Anaplasmataceae genes. These included 37 *p44* pseudogenes, five *HGE-14* paralogs (HGE1_01782, 01752, 01772, 02100, and 02107 in descending order of transcript signal abundance), and HGE1_05792, an msp2 family gene (Figures S2, S7). These genes encode bacterial cell surface proteins (P44 and Msp2 family) and putative type 4 secretion system (T4SS) effectors that are translocated into the HL-60 cell nucleus [*HGE-14*; (21)]. Hypothetical protein genes were the next most numerous (32) category in HL-60 cells (Table S1A). Hypothetical proteins were assessed using three computational models: the SecretomeP 2.0 Server (secP) (32), which predicts non-signal-peptide-triggered protein secretion; the SignalP-5.0 Server (sigP) (33), which predicts the presence of signal peptides; and CELLO, a subcellular localization predictor (34). Fifteen of the 32 were predicted by secP as secreted or by CELLO as localized to the bacterial cell membranes, periplasm, or as extracellular (none were by sigP) (Table S1A). HGE1_00140, the most upregulated gene (hypothetical) in HL-60 (5.9-fold), is localized to the morulae membrane late in HL-60 cell infection (18). Surface protein gene, HGE1_05792, which encodes an MSP2 family outer membrane protein, was upregulated during replication in HL-60 cells (sample 6), but not in ISE6 cells (sample 7; Figure S2). Next in abundance were 11 genes annotated by Clusters of Orthologous Groups (COGs) as translation, ribosome structure and biogenesis (vs. two in ISE6), followed by four genes each in COGs categories transcription and energy production and conservation (vs. none in either in ISE6). Another 20 genes with elevated transcript levels in HL-60 have diverse or unknown functions (Table S1B). Twenty-five of 42 genes displaying increased transcript levels in ISE6 cells were hypothetical genes, 15 of which were predicted to be secreted or membrane associated. Also upregulated in ISE6 cells were four genes with roles in replication, recombination and repair (vs. one in HL-60), and the transcription regulator *tr1* (HGE1_05312), which is the first of four genes that constitute the *p44* expression site (*p44*ES) (20). *tr1* and a *p44* paralog, HGE_05367, located just downstream were specifically and significantly upregulated in Sample 3 (Figure 3), as indicated by signal corresponding to the center hypervariable region, but none of the other *p44* pseudogenes were (Table S1B).

Comparison of samples taken during replication of Ap in human granulocytes (24 h) (Figure 5C, Giemsa-stained) to those of Ap replicating in HL-60 cells (Figure 5A) showed similar profiles over large sections of the genome (Figure 3), but also identified 225 differentially transcribed genes (≥1.5-fold elevated, *p* ≤ 0.05), 105 with elevated mRNA levels in HL-60 cells and 120 in granulocytes (Table S2A). Genes elevated in HL-60 cells relative to granulocytes included 28 with roles in translation, ribosomal structure and biogenesis (vs. nine in granulocytes); 10 that encode tRNA (vs. one in granulocytes); six unique Anaplasmataceae genes (five *p44* pseudogenes and HGE1_06087 (Figure S3); six with roles in nucleotide transport and metabolism (vs. three in granulocytes); five with roles in lipid transport and metabolism (vs. two in granulocytes); four involved in cell wall/membrane/envelope biogenesis (vs. one in granulocytes); and four genes that participate in transcription (vs. one in granulocytes; Table S2B). The disproportionate upregulation of genes in these categories suggests an emphasis on bacterial growth in HL-60. An exception is HGE1_06087, a unique Anaplasmataceae gene encoding a 100-kDa immune-dominant antigen, HGE-2, that has been shown to be surface-expressed on bacteria within morulae and on the morulae membrane (18), is also upregulated in HL-60 cells (Figure S3). Over-represented genes in granulocytes included 40 hypothetical genes (vs. 15 in HL-60), 10 unique Anaplasmataceae genes (seven *p44* pseudogenes and three msp2 paralogs), and nine genes each in the following COGs categories: coenzyme transport and metabolism (vs. three in HL-60), post-translational modification, protein turnover, chaperones (vs. three in HL-60), energy production and conversion (vs. three in HL-60), and intracellular trafficking, secretion, and vesicular transport (vs. two in HL-60; Table S2B). The transcription regulator *tr1* was upregulated 3.2-fold in granulocytes and several full-length *p44* pseudogenes were upregulated in both cell types. CELLO, secP, and sigP analyses of hypothetical proteins predicted that 17 of those elevated in granulocytes and seven of those elevated with HL-60 cells are either secreted (by secP) or localized to the bacterial cell membranes or periplasm (Table S2A).

### *P44* Hypervariable Region Signals

Antigenic variation in Ap is mediated by recombination within the HVR of P44 transcribed from the specific *p44* copy located in the expression site (35). The 93 *p44* pseudogenes that occur in the genome of Ap isolate HGE1 differ primarily in their hypervariable region (HVR) sequences (bound by LAKT residues, depicted yellow in Artemis plots), which often showed little corresponding signal even when signal from the whole pseudogene (including from conserved sequences flanking the HVR) met the threshold for being designated as differentially transcribed. Actually transcribed *p44* HVRs with their associated anti-sense signals are illustrated in Figure 2 and listed in Table S9.

As with the whole gene measurement method, the HVR only ratings indicated that *p44* transcription levels were highest after 2 h with ISE6 and at 24 h in HL-60 (average HVR signal levels were 1.7 for each). This evaluation of HL-60 and ISE6 specific HVRs revealed a spatial pattern of *p44* pseudogene usage. In HL-60, the HVRs of *p44-37* and *p44-37b*, which are essentially duplicates, displayed strong signals from 2 h after exposure to fresh host cells through 24 h. A *p44* that has been identified as expressed during infection of HL-60 (36, 37), *p44-18*, showed prominent HVR signals at 2 h in the presence or absence HL-60 cells, and at 24 h during replication in HL-60. In ISE6, the HVR of *p44-65* showed a strong signal at 2 h (vs. none at 2 h with HL-60), and the HVRs of HGE1_04365, *p44-62, p44-26, p44-51*, and *p44-11*, all had strong signals, especially at 4 h and 48 h.

*P44* genes nearer the genomic origin of replication (toward the top and bottom of the sequential *p44* list in Table S9) tended to display higher HVR signals, and a run of seven (HGE1_04617-04807) further from the origin showed very little transcription signals in all three host cell types. This pattern of *p44* pseudogene usage by Ap is in agreement with findings by Foley et al. (38), although pseudogenes closer to the *p44*ES were no more likely to occupy it than more distant ones (Table S9). Ap infecting human granulocytes at 24 h showed an HVR transcription profile most similar to that of infected HL-60 cells at 24 h, whereas HL-60-grown bacteria incubated with ISE6 cells 2 h (Sample 5) had shifted to a *p44* profile resembling that in Sample 3 (ISE6-grown bacteria exposed to ISE6 cells 2 h).

## Discussion

The near lack of differential transcription between sample 1 and bacteria incubated for the same period by themselves suggested that cell-free bacteria either did not alter their gene transcription in response to contact with uninfected HL-60 cells, or that 2 h of co-incubation with uninfected host cells was insufficient time to detect a shift in transcription. HL-60-grown Ap bacteria exposed to fresh HL-60 cells for 2 h (sample 1) upregulated only three genes relative to control bacteria in medium alone (Table S3A), and after 4 h (sample 2), there were no differences with control bacteria. This failure of host cell contact to induce substantial new bacterial transcription suggested that, upon release from parent host cells, those genes required for invasion of new HL-60 cells were already expressed. The shift to ISE6-specific genes by HL-60-grown bacteria exposed to ISE6 cells in Sample 5 (Table S5A) indirectly supported this conclusion. Moreover, it demonstrated an induced bacterial response and suggested that tick cell membranes are sufficiently different to require retooling of the bacterial membrane to facilitate cell attachment and invasion. In HL-60 cells and human granulocytes, P-selectin glycoprotein ligand 1 (PSGL-1) is the receptor mediating infection by Ap (4), which is absent in arthropods where sialylated glycoproteins are rare. Overall, the arthropod glycome is quite different from that of mammals (39), and arthropod-borne pathogens thus utilize different cell surface moieties to colonize the vector vs. the mammalian host, although basic strategies are conserved (40).

In contrast, Ap bacteria from ISE6 tick cells had a strong transcriptional response to contact with uninfected tick cells (Sample 3, Table S4A), displaying 48 genes that were upregulated in comparison to the control (2 h in medium). These included several genes that encoded structural components of the type 4 secretion system (T4SS) of Ap (two *virB2*, one *virB6*), or were predicted to be effectors, i.e., HGE1_01777, an *HGE-14* paralog, and six hypothetical genes (21, 22). The specific ISE6 host components that these putative effectors interact with are unknown, but it is predictable based on the divergent biology and structure of tick vs. human cells that Ap should be using specific effectors for vector and human host cells. Among 17 upregulated genes associated with early infection of ISE6, there were four hypothetical genes (HGE1_02797, 03277, 05647, and 02747) (Table S5A). The 17 genes were found to be upregulated both in ISE6-grown bacteria incubated with ISE6 cells (sample 3) and in HL-60-grown bacteria incubated with ISE6 cells (Sample 5), when each was compared to Samples 1 and 2 (HL-60-grown bacteria incubated with HL-60 cells). Two of them (HGE1_05647 and HGE1_02122) had elevated mRNA levels in sample 3 compared to sample 7, suggesting that their role was restricted to invasion, while three others had elevated transcript levels in Sample 7 (Table S8) including HGE1_03797 (27-fold elevated). This indicated that their gene products had roles primarily in subsequent intracellular replication. The strongly upregulated HGE1_03797 encodes a hypothetical protein predicted by CELLO to localize to the bacterial cytoplasm and by secP to be secreted, which reflects our lack of understanding how Ap proteins function.

The conserved flanking regions of 50 *p44* pseudogenes displayed elevated transcript levels in sample 3 (2 h with ISE6) compared to sample 4 (4 h; Table S4C), but only three did in comparison to the 2 h control (Table S4A). This phenomenon reflected upregulation of a subset of *p44* genes in which HVRs showed transcript signals at the same time, and not of all of them. Apparently, *p44* transcript levels were already high in the control, likely upon release from parent ISE6 cells. By 4 h of co-incubation with ISE6 host cells (sample 4), the number of upregulated Ap genes had declined to eight, while in controls (bacteria incubated alone in medium), elevated transcript levels were still seen in 37, including conserved regions of many *p44*s (Table S4B), indicating a transition away from cell-specific transcription. The decreased *p44* transcript levels after 4 h incubation with ISE6 vs. their elevation in the 4 h control suggested that cell contact led to the decrease. Research with the related *A. marginale* bovine anaplasmosis agent similarly demonstrated that the diversity of *msp2* sequences in the expression site of bacteria harvested from tick cells was reduced in comparison to bacteria harvested from blood or from mammalian cell lines (41). This was taken to indicate that the type of MSP2 produced changed in response to immune pressure (which is more complex in cattle than in ticks) as well as during invasion of different host cells that present diversities in membrane structure (42, 43).

The apparent transcription of 50 *p44* pseudogenes in ISE6 tick cell-grown Ap after 2 h incubation with fresh ISE6 (Table S4C) and of 37 in Ap replicating in the human cell line (Table S1) is striking. However, transcription data showed that many of them had elevated signals corresponding to the conserved ends of the pseudogenes only, but not to intervening hypervariable regions (HVRs). This suggested that they were not transcribed from the expression site (*p44*ES), consistent with our previous study (7). Conserved sequence transcripts of all bacteria in the population generated from pseudogenes occupying the *p44*ES likely hybridized not only to probes corresponding to the specifically transcribed pseudogenes but to all probes that were homologous (those representing conserved sequences of other *p44*s), giving an inflated impression of *p44* transcription diversity. The appearance of *p44* fragments in differential gene transcription lists is further evidence of this. In HGE1, 13 of these truncated *p44*s consist of only conserved sequences, and their elevated signals must result from hybridization to homologous transcripts from specifically transcribed pseudogenes. Thus, the apparent transcription of numerous *p44* pseudogenes may be better understood as increased transcription of fewer pseudogenes, which were identified through HVR signal assessment (Table S9). However, the existence of duplicate pseudogenes, such as *p44-37* and *-37b*, which both showed strong HVR signals in HL-60, as well as other HVRs with significant sequence identity, means care must be taken in interpreting even HVR signals. Possibly only either *p44-37* or *-37b* was specifically transcribed, with signal from the other the result of cross hybridization, as in the conserved sequences above. Our data presented here are consistent with results demonstrating differential transcription of *p44*s in the tick vector and in different hosts with specific immune defects, as well as *in vitro* (44). Such studies reinforce the idea of molecular adaptation of Ap conferred by P44 to colonize its tick vector and its mammalian hosts, and of P44-mediated immune evasion.

*P44* acts as a porin thought to allow diffusion of metabolic intermediates from the host cell into the bacterium (45), with the HVR providing antigenic variation for immunoevasion. A further role in cell binding is suggested by the host cell specific expression of particular pseudogenes that have been identified in infected cell lines previously (36, 37) and here in HL-60 and ISE6. The upregulation of *p44*s during Ap replication in HL-60 cells (sample 6; Figure S3) suggested that the porin function was called for, and by ISE6-grown bacteria after 2 h incubation with fresh ISE6 cells (sample 3), that a function in cell binding was important for the largely extracellular bacteria (Figure 3, Figure S5). Spatial clustering within the genome of specifically transcribed *p44*s (with elevated HVR signals) around the origin of replication is consistent with the findings of Foley et al. (38), and the run of seven barely transcribed *p44*s is an interesting example of genomic location correlating with a lack of use. The overrepresentation of conserved sequence transcripts is reminiscent of the antisense signals seen corresponding to conserved *p44* sequences in Artemis plots of the data (Figures 2, 3), which, given their particular prominence in ISE6-grown Ap, may serve to keep *p44* expression low.

In general, there was a negative relationship between *tr1* (HGE1_05312) activity and *p44* transcription in Ap derived from ISE6 tick cells when interacting with uninfected tick cells, suggesting that this transcriptional regulator dampened *p44* transcription under these circumstances. In HL-60-grown Ap incubated 2 h with fresh HL-60, *apxR*, a regulator of *tr1* linked to increased *p44* transcription in mammalian cells (23, 24), was upregulated 4.8-fold relative to the 2 h ISE6 sample (Table S6A). However, *p44* transcription appears to be greater at 2 h with ISE6 (Tables S6A, S9) instead of at 2 h with HL-60, and *tr1* is upregulated 9.1-fold in the ISE6 sample. This counter-intuitive situation—*apxR* elevated but *p44* low (2 h in HL-60) and both *tr1* and *p44* elevated (2 h in ISE6)—hints at a more complex regulatory process behind *p44* transcription.

The overall greater differential transcription seen in Ap incubated with ISE6 when comparing the 2 h time-point to the 4 h time-point (167 genes at 2 h, 65 at 4 h) than was evident in comparisons to controls (48 genes at 2 h, 8 genes at 4 h) (Tables S4A–S4C), suggests that much of the change occurred as a function of time rather than in response to cell contact. This was essentially entirely the case with HL-60 in which 45 bacterial genes were upregulated at 4 h in comparison to 2 h (Table S3B), but none were in comparison to the 4 h control. Nonetheless, when compared to bacteria replicating in HL-60 (Table S7) or binding/entering ISE6 (Table S6), numerous genes with elevated transcript levels were identified in the 2 h HL-60 sample, including five *HGE-14* paralogs (HGE1_01782, 01772, 01752, 02107, and 02100) in both comparisons and with transcript levels in the same order by abundance. These paralogs were also upregulated, and in a similar order of transcript abundance (HGE1_01782, 01752, 01772, 02100, and 02107), in HL-60 replication samples relative to ISE6 replication samples, suggesting a role specifically in manipulating the mammalian host. Bacteria binding/entering ISE6 cells for 2 h upregulated their own *HGE-14* paralogs: HGE1_02092 in comparison to bacteria incubated 2 h with HL-60 (Table S6); and HGE1_01777 in comparison to the control (Table S4A), and in Sample 5 in comparison to 2 h HL-60 (Table S5). Thus, five particular *HGE-14* paralogs (of ten that are annotated and an eleventh that is indicated by transcription plots, Figure S7) were upregulated at consistent individual levels in bacteria binding/entering and replicating in HL-60, as were two others in bacteria binding/entering ISE6 cells. The suggestion is that these nucleus-targeting effectors are specific to mammalian or tick host cells and in human cells operate collectively but with discrete functions. Since no *HGE-14* paralogs were differentially transcribed in the 24 h HL-60/24 h granulocyte comparison, it can be inferred that the five were transcribed at similar levels in granulocytes. The expression of fewer *HGE-14* paralogs in ISE6 than human cells may reflect the lower complexity of the tick host system that requires less manipulation of a more primitive immune system for Ap to infect and thrive.

Other notable genes with elevated transcript levels in bacteria binding/entering HL-60, both in comparison to those replicating in HL-60 (Table S7) and those binding/entering ISE6 cells (Table S6), included: HGE1_05392 (product predicted to be secreted), shown to be expressed by infectious organisms (10); HGE1_00140 (product predicted to be secreted), identified on the morulae membrane (18); HGE1_01550, encoding DnaK, which localizes to the bacterial membrane (28); HGE1_03902, encoding AipA, an invasin of mammalian cells (25); and HGE1_01090, encoding a protein expressed on the bacterial surface and required for infection (31). Upregulation of these genes encoding surface-expressed or secreted proteins with roles in cell infection was specifically associated with HL-60 cell binding and entry in this study, consistent with the studies mentioned.

As in the tick cells, a set of bacterial genes that encode hypothetical proteins was specifically upregulated in bacteria binding/entering the human cell line. In comparison to bacteria either replicating in HL-60 or binding/entering ISE6, 18 hypothetical genes were upregulated in bacteria binding/entering HL-60 cells, 15 of which were predicted to be secreted or localized to the bacterial membrane or periplasm (Table S7). These bacterial-membrane-associated proteins as well as those encoded by genes upregulated early during ISE6 cell infection, likely facilitate host cell binding, invasion, and immune avoidance in ways unique to Ap, and are thus of particular interest for functional studies.

Bacteria replicating in HL-60 vs. ISE6 displayed more genes involved in translation, ribosome structure and biogenesis (11 vs. 2 in ISE6), transcription (4 vs. 1 in ISE6), energy production and conversion (4 vs. 0 in ISE6), and lipid transport and metabolism (3 vs. 0 in ISE6; Table S1B), suggesting that comparatively robust synthetic processes were underway in the human cells. This may be a reflection of the slower growth of Ap in the tick cells, which require about 2 weeks to become maximally infected compared to 4 days in HL-60.

In human granulocytes vs. HL-60 cells, bacterial transcription showed major differences in three gene categories: translation, ribosomal structure, and biogenesis (28 genes upregulated in HL-60 vs. 9 in granulocytes); hypothetical genes (15 in HL-60 vs. 40 in granulocytes); and transfer RNA genes (10 in HL-60 vs. 1 in granulocytes; Table S2B). The comparatively high tRNA transcript levels in bacteria infecting HL-60 cells included the six most differentially transcribed genes (4.4- to 7.6-fold elevated, Table S2A), and in conjunction with no differences in tRNA levels between HL-60 and ISE6 replication samples (Table S1A), indicated that bacterial tRNA levels in granulocytes were especially low. Transfer RNA genes were also relatively upregulated at 2 h with ISE6 compared to 4 h (Table S4C), and at 4 h with HL-60 compared to 2 h (Table S3B). Aside from their obvious role in protein biosynthesis, prokaryotic tRNAs have diverse functions encompassing regulation of gene expression including suppression, and as substrates for cell wall formation, protein degradation, aminoacylation of phospholipids in cell membranes, and antibiotic biosynthesis [reviewed by Raina and Ibba (46) and Shepherd and Ibba (47)].

In this study, tRNA transcription was low in the primary host cells (human granulocytes), but high in the cell lines, consistent with the observation that in humans, relatively few granulocytes are infected that support limited Ap growth and replication. Differentiated neutrophil granulocytes secrete branched-chain, aromatic and positively charged free amino acids upon adhesion to extracellular matrix (48), and inhibition of this response may increase availability to intracellular Ap (49). Evidence derived from *in vitro* studies of Ap and also *in vivo* with the related *Anaplasma marginale* indicates that microvascular endothelial cells are a nidus of infection (50–52), and these long-lived cells likely offer an environment supportive of growth and replication that requires import of amino acids. This and the substantial increase in hypothetical genes upregulated in granulocytes illustrates how little we understand of the interaction between Ap and granulocytes and highlights the need for follow-up testing *in vivo* or with primary cells. Other differences in Ap transcription between granulocytes and HL-60 cells included three times as many upregulated genes in granulocytes with roles in coenzyme transport and metabolism, post-translational modification, protein turnover, chaperones, energy production and conversion, and intracellular trafficking, secretion, and vesicular transport. This shows that Ap is very active during its residence in these cells, re-programming cellular defense responses to turn these professional phagocytes that predominantly utilize glycolysis for generation of energy (53) into a supportive host cell (54, 55).

We noted that direct labeling of total RNA from Ap-infected cells produced strong, specific signals that corresponded to the genomic source and relative abundance of all Ap mRNA transcripts on a whole genome tiling array. In addition to providing transcript levels for all predicted bacterial genes during the earliest stage of human and tick cell infection and proliferation in both cell lines and human granulocytes, specific transcription peaks indicated the presence of unannotated genes and antisense transcripts that may play roles in host cell infection. Genes that encode hypothetical proteins that are unique to Ap and particularly enable the pathogen to manipulate tick and mammalian host cells are key to understanding how those processes work and can themselves be manipulated to prevent infection. Current work in our laboratory and others with mutant libraries containing single-insertion mutants, many into genes encoding hypothetical proteins, will be guided by these data, which identify the genes (the mutants) to prioritize in phenotypic screens designed to reveal gene function.

## Conclusions

The gene expression profiles of Ap isolate HGE1 during invasion and replication in cell cultures from its vector and human host reflected its need to adapt to these biologically divergent hosts. Analysis of gene activation in Ap from human promyelocytes (HL-60) exposed to uninfected HL-60 cells showed little change, and the bacteria appeared pre-programmed for invasion of the same mammalian host cell. By contrast, the expression profiles of HL-60 grown or tick cell (ISE6) culture-derived Ap when incubated with uninfected ISE6 cells, demonstrated significant new gene upregulation, suggesting Ap were primed for multiple rounds of infection and invasion of mammalian cells, but not tick cells. These results indicated that Ap recognized the host species cell they are exposed to, and adapted quickly, a conclusion further supported by upregulation of host-cell specific genes encoding effectors and T4SS components that would facilitate control of host cell transcription. Remarkably, Ap gene expression in human granulocytes *ex-vivo* included a large set of hypothetical genes and genes in categories related to production of proteins and metabolites, but was otherwise similar to that seen in HL-60 cells. This is consistent with the fact that HL-60 cells replicate in culture, while granulocytes do not. With our research, many hypothetical genes are now linked to specific events during the life cycle of Ap, which provides important clues to their function. Given the significant involvement of hypothetical gene products in important Ap functions, such as replication in human neutrophils, which is linked to pathogenicity of anaplasmosis, it would be very rewarding to determine their function.

## Data Availability Statement

Raw microarray data have been deposited in the Dryad database, available at https://doi.org/10.5061/dryad.18931zcs5.

## Ethics Statement

The studies involving human participants were reviewed and approved by the Internal Review Board of the University of Minnesota. The patients/participants provided their written informed consent to participate in this study.

## Author Contributions

CN designed the custom tiling array, designed and carried out the study, and wrote the manuscript. MH normalized the microarray data, performed the statistical analyses, and prepared the data for viewing in Artemis. X-RW did the confocal microscopy and assisted with preparing figures. GB provided the editorial assistance with the manuscript. JO helped to interpret the results and submitted the data sets to the Dryad repository for sharing with other researchers. UM was the principal investigator, oversaw the study and assisted with writing the manuscript.

### Conflict of Interest

The authors declare that the research was conducted in the absence of any commercial or financial relationships that could be construed as a potential conflict of interest.

## Supplementary Material

Figure S1 Blue bars (arrows) represent *A. phagocytophilum* transcription signals detected with Cy3-labeled cDNA in sample 6 (24 h in HL-60 cells), using the FairPlay III kit from Agilent. Actinomycin D was added in an attempt to prevent spurious second strand cDNA synthesis. Black bars represent *A. phagocytophilum* transcription signals detected using the same sample 6 material in which RNA was directly Cy3-labeled via the Kreatech kit (Leica Biosystems). Turquoise boxes below the transcript plots denote coding regions of annotated genes. Each bar corresponds to one 60-mer probe on the tiling array representing positive (upper panel) and negative (lower panel) strands of the genome between genes HGE1_04417 and *tolB*. Direct labeling avoids reverse transcriptase-mediated production of cDNA, which may continue to produce second strand cDNA that hybridizes to anti-sense DNA (blue bars, lower plot). The direct labeling method (black bars) produced stronger transcription signals without the false antisense signals seen with the indirect method. Labeled cDNA or RNA from sample 6 was hybridized to arrays contained on the same 8-plex slide and the data were not normalized. Subsequently, only directly labeled RNA from all samples was used. Nucleotide positions in the genome are indicated by numbers above the gray line.

Figure S2 Transcription data (top two graphs) plotted over the annotated genome sequence (sections at the bottom) using the Artemis genome browser. Turquoise boxes denote coding regions of annotated genes, and pink boxes denote *p44* conserved sequences with yellow boxes denoting their center hypervariable region. Each bar corresponds to one 60-mer probe on the tiling array. Black bars represent Ap transcription in HL-60 cells at 24 h (sample 6), red bars represent Ap transcription in ISE6 cells at 48 h (sample 7). HGE1_05792, which encodes an MSP2 family outer membrane protein, is upregulated during replication in HL-60 cells (sample 6), but not in ISE6 cells (sample 7). Three *p44* paralogs (HGE1_05805, HGE1_05812, HGE1_05817) show characteristic signals associated with their conserved ends (pink) but little specific signal corresponding to their hypervariable regions (yellow). Nucleotide positions in the genome are indicated by numbers above the gray line.

Figure S3 Transcription data (top two graphs) plotted over the annotated genome sequence (sections at the bottom) using the Artemis genome browser. Turquoise boxes denote coding regions of annotated genes, and pink boxes denote *p44* conserved sequences with yellow boxes denoting their center hypervariable region. Each bar corresponds to one 60-mer probe on the tiling array. Black bars represent Ap transcription in HL-60 cells at 24 h (sample 6), and blue bars represent Ap transcription in human granulocytes at 24 h (sample 8). HGE1_06087, a unique Anaplasmataceae gene encoding HGE-2 that is surface-expressed on bacteria within morulae and on the morulae membrane (18), is upregulated in HL-60 cells. Two *p44* paralogs, HGE1_06097 and 06102, have strong transcription signals associated with their conserved ends (pink boxes) but not their hypervariable regions (yellow boxes), while two others (HGE1_06127 = p44-37, HGE1_06132 = p44-37b) show strong signals that include their hypervariable regions, indicating transcription from the *p44* expression site by a substantial percentage of the bacteria. All *p44* paralogs show greater transcription in HL-60 cells than in human granulocytes. Nucleotide positions in the genome are indicated by numbers above the gray line.

Figure S4 Transcription data (top two graphs) plotted over the annotated genome sequence (sections at the bottom) using the Artemis genome browser. Turquoise boxes denote coding regions of annotated genes. Each bar corresponds to one 60-mer probe on the tiling array. Black bars represent Ap transcription at 2 h with HL-60 cells (sample 1), red bars represent Ap transcription at 2 h with ISE6 cells (sample 3). Each bar corresponds to one 60-mer probe on the tiling array. HGE1_03552, a hypothetical gene, is strongly upregulated in sample 3, but not in sample 1. Another hypothetical gene, HGE1_03512, is upregulated at 2 h in sample 1, but not in sample 3. Antisense signals, especially from sample 3 (red bars), can be seen opposite sense transcription signals from several of the genes. Also of note is an unannotated peak just upstream of HGE1_03517. Nucleotide positions in the genome are indicated by numbers above the gray line.

Figure S5 Transcript signals from Samples 1 and 3 in *A. phagocytophilum* (Ap) strain HGE1. Transcription data (top two graphs) plotted over the annotated genome sequence (sections at the bottom) using the Artemis genome browser. Turquoise boxes denote coding regions of annotated genes, and pink boxes denote *p44* conserved sequences with yellow boxes denoting their center hypervariable region. Each bar corresponds to one 60-mer probe on the tiling array. Black bars represent Ap transcription at 2 h with HL-60 cells (sample 1), red bars represent Ap transcription at 2 h with ISE6 cells (sample 3). Transcription regulator 1 (*tr1*) (HGE1_05312) is upregulated only in sample 3, and is positioned at the beginning of the *p44* expression site (p44ES) (19), which includes *omp-1x* (HGE1_05317), *omp-1N* (HGE1_05322), and the particular *p44* paralog being expressed (HGE1_05327) all polycistronically transcribed (20). Notably, recombinase A (HGE1_05332) is co-transcribed in both tick and human cells, suggesting a possible role in *p44* recombination. Nucleotide positions in the genome are indicated by numbers above the gray line.

Figure S6 Transcription data (top two graphs) plotted over the annotated genome sequence (sections at the bottom) using the Artemis genome browser. Turquoise boxes denote coding regions of annotated genes. Each bar corresponds to one 60-mer probe on the tiling array. Green bars represent Ap transcription in sample 1 (2 h with HL-60), black bars represent Ap transcription in sample 6 (24 h in HL-60). HGE1_01020, a hypothetical gene whose product is predicted by CELLO to localize to the bacterial inner membrane, and groEL, which is translocated into host cell nuclei (21), are upregulated in sample 1, suggesting that Ap may alter host cell responses even before or immediately after host cell invasion. Nucleotide positions in the genome are indicated by numbers above the gray line.

Figure S7 Transcription data (top two graphs) plotted over the annotated genome sequence (sections at the bottom) using the Artemis genome browser. Turquoise boxes denote coding regions of annotated genes. Each bar corresponds to one 60-mer probe on the tiling array. Green bars represent Ap transcription in sample 1 (2 h with HL-60), black bars represent Ap transcription in sample 6 (24 h in HL-60). Three annotated HGE-14 genes (HGE1_01752, HGE1_01772, and HGE1_01782) and one on the same DNA strand between HGE1_01752 and HGE1_01772 that is not annotated are upregulated at 2 h with HL-60. HGE-14 proteins are putative effectors predicted to enter the host nucleus and alter transcription (21, 22). Nucleotide positions in the genome are indicated by numbers above the gray line.

Figure S8 Transcription data (top two graphs) plotted over the annotated genome sequence (sections at the bottom) using the Artemis genome browser. Turquoise boxes denote coding regions of annotated genes. Each bar corresponds to one 60-mer probe on the tiling array. Green bars represent Ap transcription in sample 3 (2 h with ISE6), red bars represent Ap transcription in sample 7 (48 h in ISE6 cells). HGE1_01872, a hypothetical gene, is upregulated in replicating bacteria. Nucleotide positions in the genome are indicated by numbers above the gray line.

Figure S9 Transcription data (top two graphs) plotted over the annotated genome sequence (sections at the bottom) using the Artemis genome browser. Turquoise boxes denote coding regions of annotated genes, and pink boxes denote *p44* conserved sequences with yellow boxes denoting their center hypervariable region. Each bar corresponds to one 60-mer probe on the tiling array. Green bars represent Ap transcription in sample 3 (2 h with ISE6), red bars represent Ap transcription in sample 7 (48 h in ISE6 cells). HGE1_05412 encoding MSP4 is upregulated during bacterial replication, whereas three *p44* paralogs (HGE1_05402, HGE1_05407, and HGE1_05427) are upregulated at 2 h with ISE6 cells.
