# Supplementary figures and images for: Global Transcription Profiles of Anaplasma phagocytophilum at Key Stages of Infection in Tick and Human Cell Lines and Granulocytes
Source: Front Vet Sci. 2020 Mar 6;7:111. doi: 10.3389/fvets.2020.00111 (PMC7069361; doi:10.3389/fvets.2020.00111)

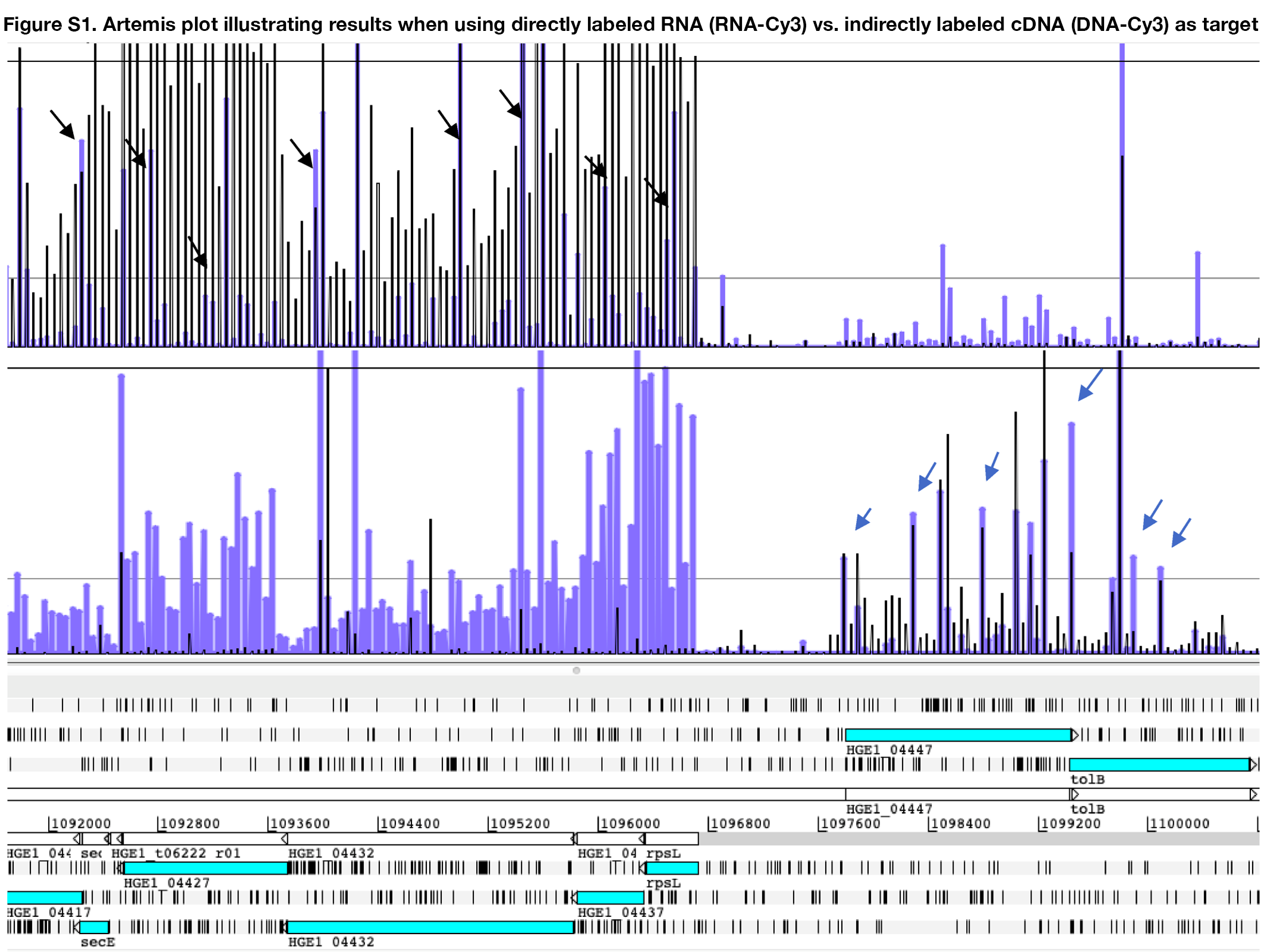

Supplement: Figure S1 — Blue bars (arrows) represent A. phagocytophilum transcription signals detected with Cy3-labeled cDNA in sample 6 (24 h in HL-60 cells), using the FairPlay III kit from Agilent. Actinomycin D was added in an attempt to prevent spurious second strand cDNA synthesis. Black bars represent A. phagocytophilum transcription signals detected using the same sample 6 material in which RNA was directly Cy3-labeled via the Kreatech kit (Leica Biosystems). Turquoise boxes below the transcript plots denote coding regions of annotated genes. Each bar corresponds to one 60-mer probe on the tiling array representing positive (upper panel) and negative (lower panel) strands of the genome between genes HGE1_04417 and tolB. Direct labeling avoids reverse transcriptase-mediated production of cDNA, which may continue to produce second strand cDNA that hybridizes to anti-sense DNA (blue bars, lower plot). The direct labeling method (black bars) produced stronger transcription signals without the false antisense signals seen with the indirect method. Labeled cDNA or RNA from sample 6 was hybridized to arrays contained on the same 8-plex slide and the data were not normalized. Subsequently, only directly labeled RNA from all samples was used. Nucleotide positions in the genome are indicated by numbers above the gray line. [file Image_1.tif]

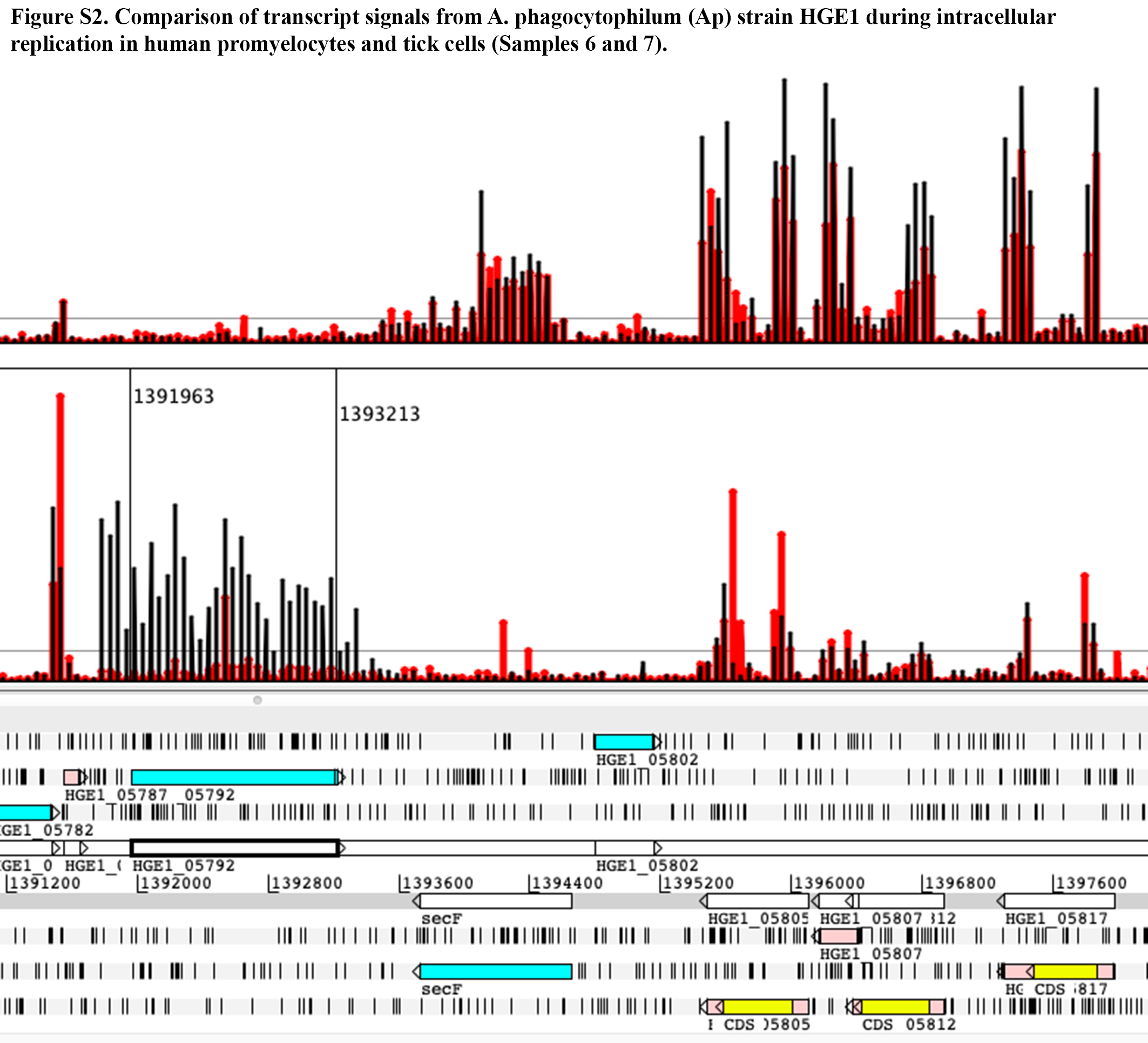

Supplement: Figure S2 — Transcription data (top two graphs) plotted over the annotated genome sequence (sections at the bottom) using the Artemis genome browser. Turquoise boxes denote coding regions of annotated genes, and pink boxes denote p44 conserved sequences with yellow boxes denoting their center hypervariable region. Each bar corresponds to one 60-mer probe on the tiling array. Black bars represent Ap transcription in HL-60 cells at 24 h (sample 6), red bars represent Ap transcription in ISE6 cells at 48 h (sample 7). HGE1_05792, which encodes an MSP2 family outer membrane protein, is upregulated during replication in HL-60 cells (sample 6), but not in ISE6 cells (sample 7). Three p44 paralogs (HGE1_05805, HGE1_05812, HGE1_05817) show characteristic signals associated with their conserved ends (pink) but little specific signal corresponding to their hypervariable regions (yellow). Nucleotide positions in the genome are indicated by numbers above the gray line. [file Image_2.tif]

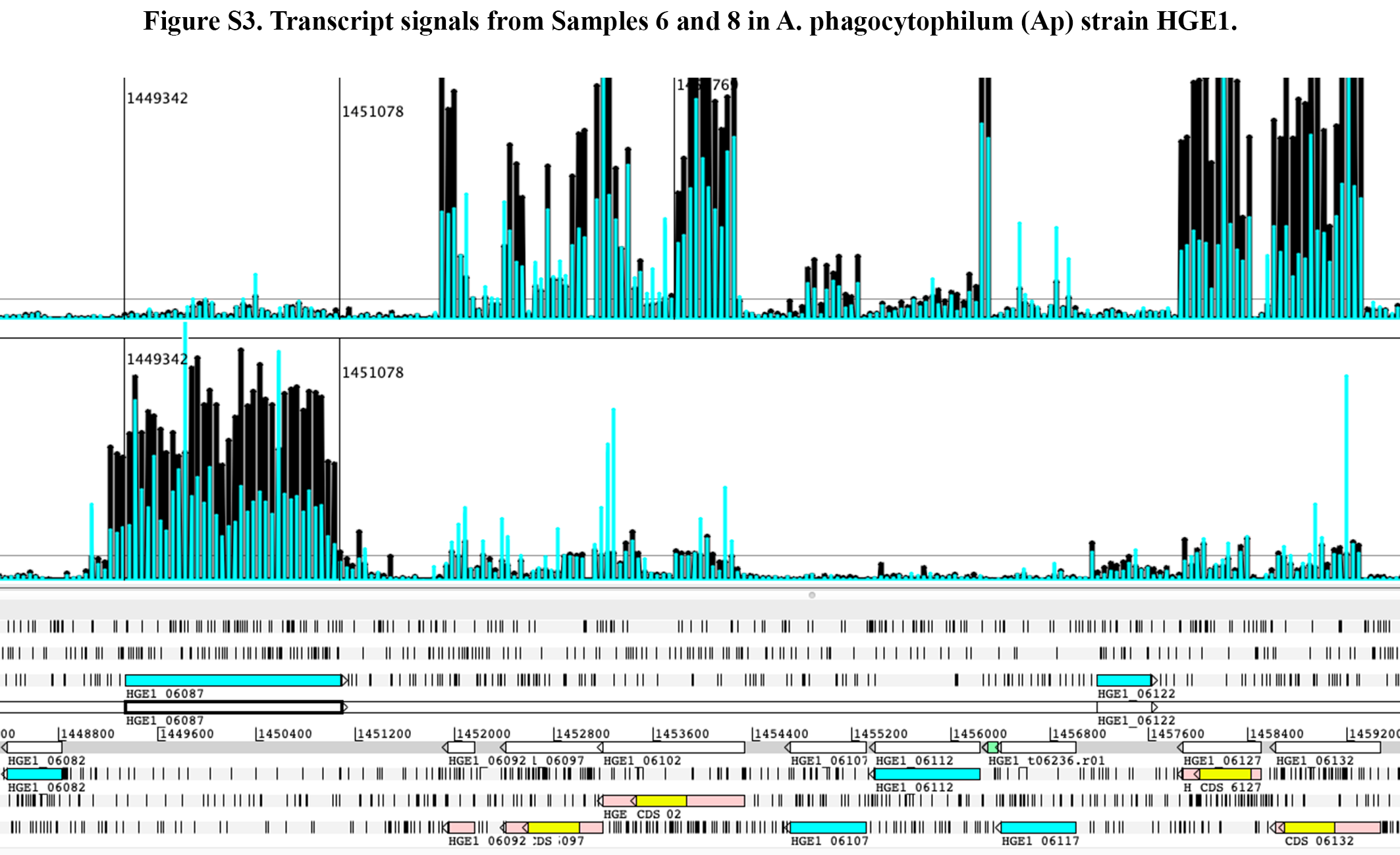

Supplement: Figure S3 — Transcription data (top two graphs) plotted over the annotated genome sequence (sections at the bottom) using the Artemis genome browser. Turquoise boxes denote coding regions of annotated genes, and pink boxes denote p44 conserved sequences with yellow boxes denoting their center hypervariable region. Each bar corresponds to one 60-mer probe on the tiling array. Black bars represent Ap transcription in HL-60 cells at 24 h (sample 6), and blue bars represent Ap transcription in human granulocytes at 24 h (sample 8). HGE1_06087, a unique Anaplasmataceae gene encoding HGE-2 that is surface-expressed on bacteria within morulae and on the morulae membrane (18), is upregulated in HL-60 cells. Two p44 paralogs, HGE1_06097 and 06102, have strong transcription signals associated with their conserved ends (pink boxes) but not their hypervariable regions (yellow boxes), while two others (HGE1_06127 = p44-37, HGE1_06132 = p44-37b) show strong signals that include their hypervariable regions, indicating transcription from the p44 expression site by a substantial percentage of the bacteria. All p44 paralogs show greater transcription in HL-60 cells than in human granulocytes. Nucleotide positions in the genome are indicated by numbers above the gray line. [file Image_3.tif]

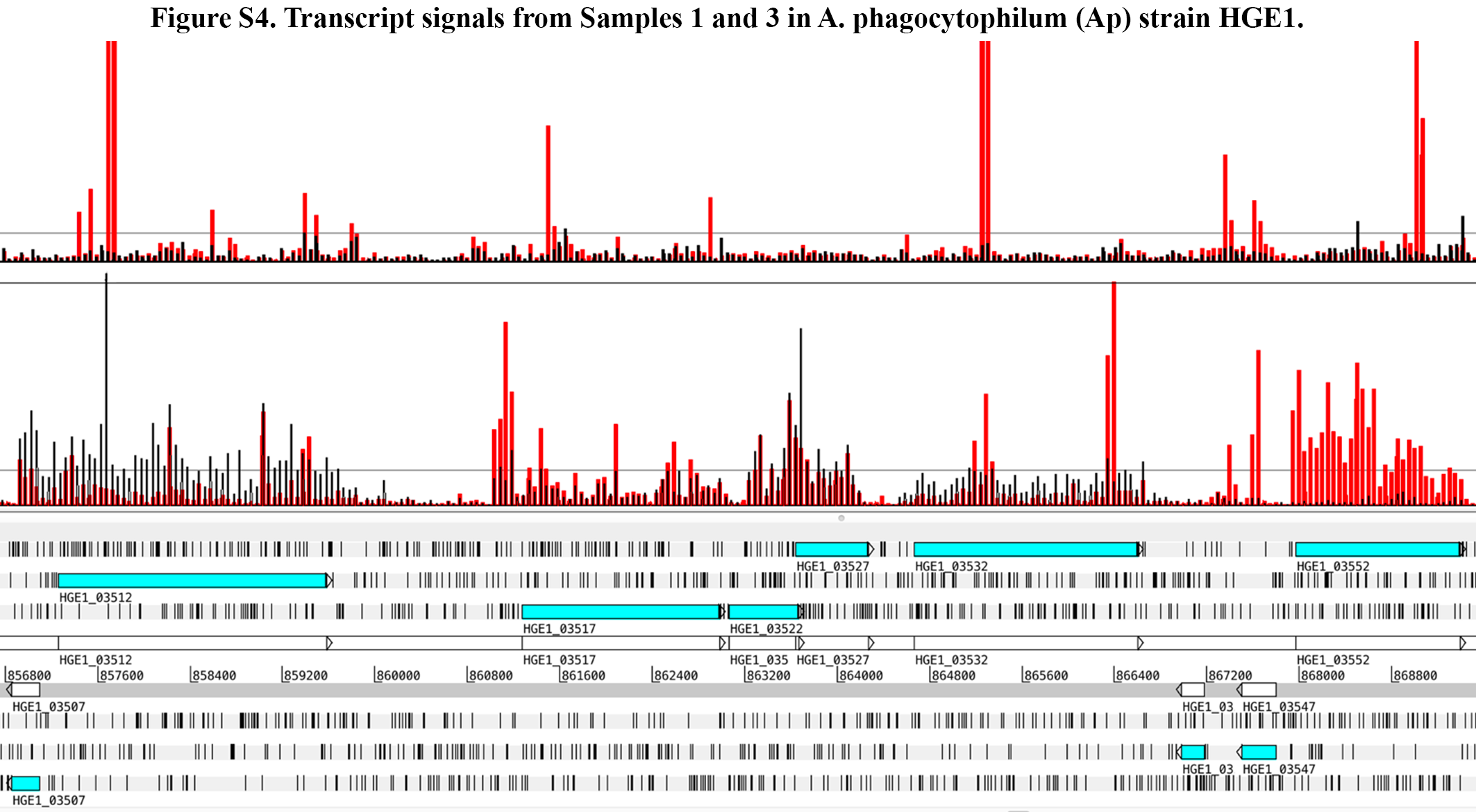

Supplement: Figure S4 — Transcription data (top two graphs) plotted over the annotated genome sequence (sections at the bottom) using the Artemis genome browser. Turquoise boxes denote coding regions of annotated genes. Each bar corresponds to one 60-mer probe on the tiling array. Black bars represent Ap transcription at 2 h with HL-60 cells (sample 1), red bars represent Ap transcription at 2 h with ISE6 cells (sample 3). Each bar corresponds to one 60-mer probe on the tiling array. HGE1_03552, a hypothetical gene, is strongly upregulated in sample 3, but not in sample 1. Another hypothetical gene, HGE1_03512, is upregulated at 2 h in sample 1, but not in sample 3. Antisense signals, especially from sample 3 (red bars), can be seen opposite sense transcription signals from several of the genes. Also of note is an unannotated peak just upstream of HGE1_03517. Nucleotide positions in the genome are indicated by numbers above the gray line. [file Image_4.tif]

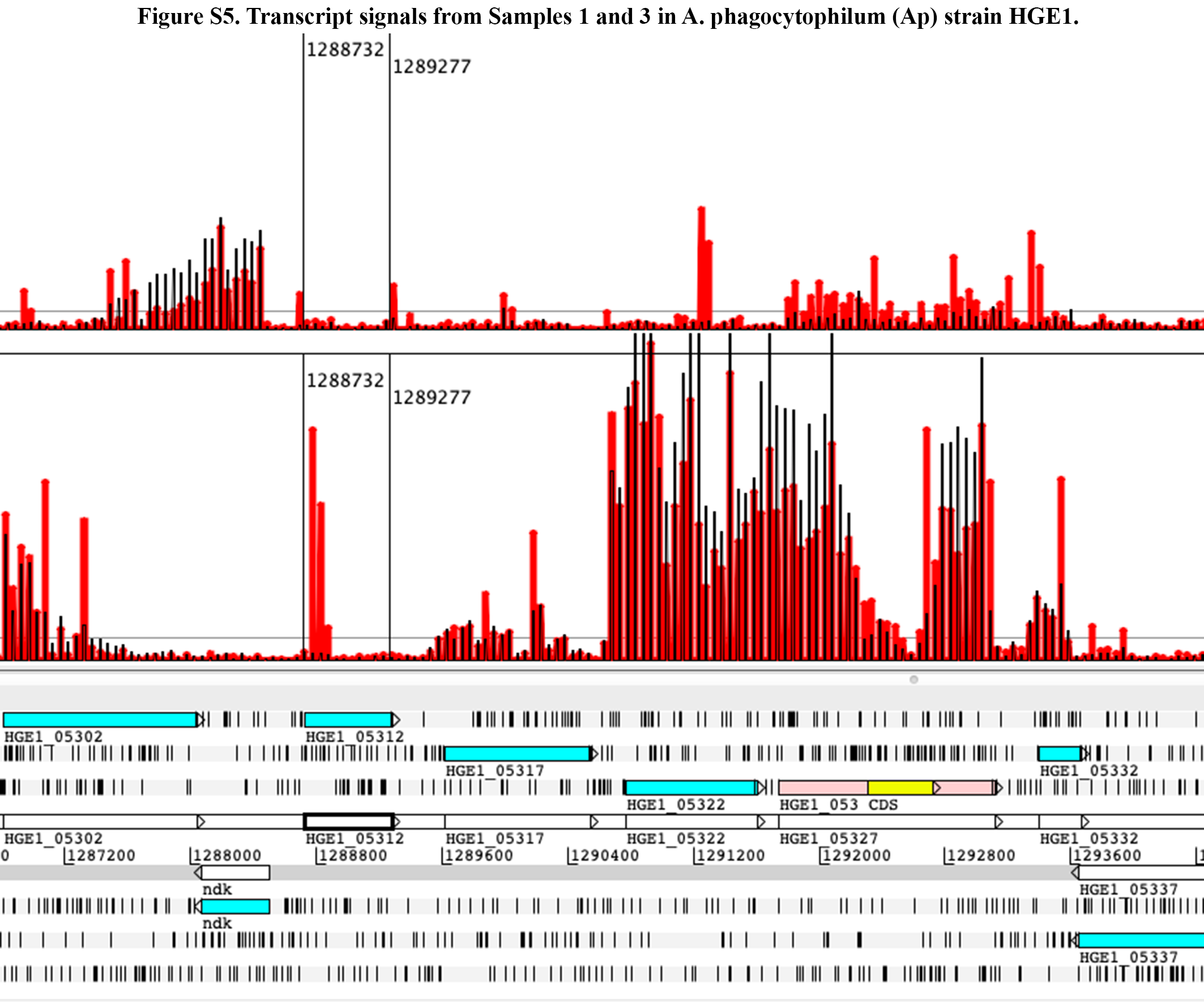

Supplement: Figure S5 — Transcript signals from Samples 1 and 3 in A. phagocytophilum (Ap) strain HGE1. Transcription data (top two graphs) plotted over the annotated genome sequence (sections at the bottom) using the Artemis genome browser. Turquoise boxes denote coding regions of annotated genes, and pink boxes denote p44 conserved sequences with yellow boxes denoting their center hypervariable region. Each bar corresponds to one 60-mer probe on the tiling array. Black bars represent Ap transcription at 2 h with HL-60 cells (sample 1), red bars represent Ap transcription at 2 h with ISE6 cells (sample 3). Transcription regulator 1 (tr1) (HGE1_05312) is upregulated only in sample 3, and is positioned at the beginning of the p44 expression site (p44ES) (19), which includes omp-1x (HGE1_05317), omp-1N (HGE1_05322), and the particular p44 paralog being expressed (HGE1_05327) all polycistronically transcribed (20). Notably, recombinase A (HGE1_05332) is co-transcribed in both tick and human cells, suggesting a possible role in p44 recombination. Nucleotide positions in the genome are indicated by numbers above the gray line. [file Image_5.tif]

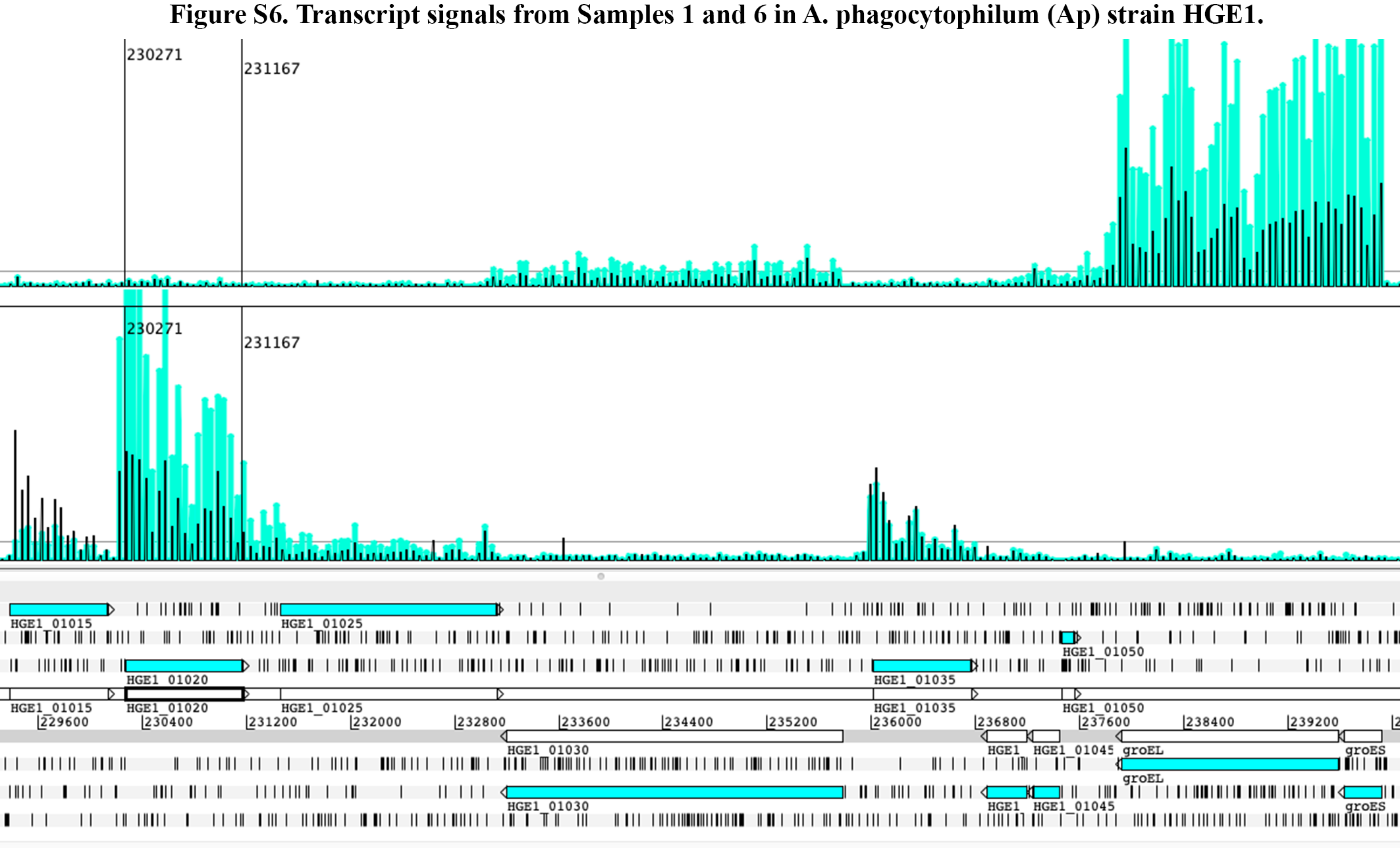

Supplement: Figure S6 — Transcription data (top two graphs) plotted over the annotated genome sequence (sections at the bottom) using the Artemis genome browser. Turquoise boxes denote coding regions of annotated genes. Each bar corresponds to one 60-mer probe on the tiling array. Green bars represent Ap transcription in sample 1 (2 h with HL-60), black bars represent Ap transcription in sample 6 (24 h in HL-60). HGE1_01020, a hypothetical gene whose product is predicted by CELLO to localize to the bacterial inner membrane, and groEL, which is translocated into host cell nuclei (21), are upregulated in sample 1, suggesting that Ap may alter host cell responses even before or immediately after host cell invasion. Nucleotide positions in the genome are indicated by numbers above the gray line. [file Image_6.tif]

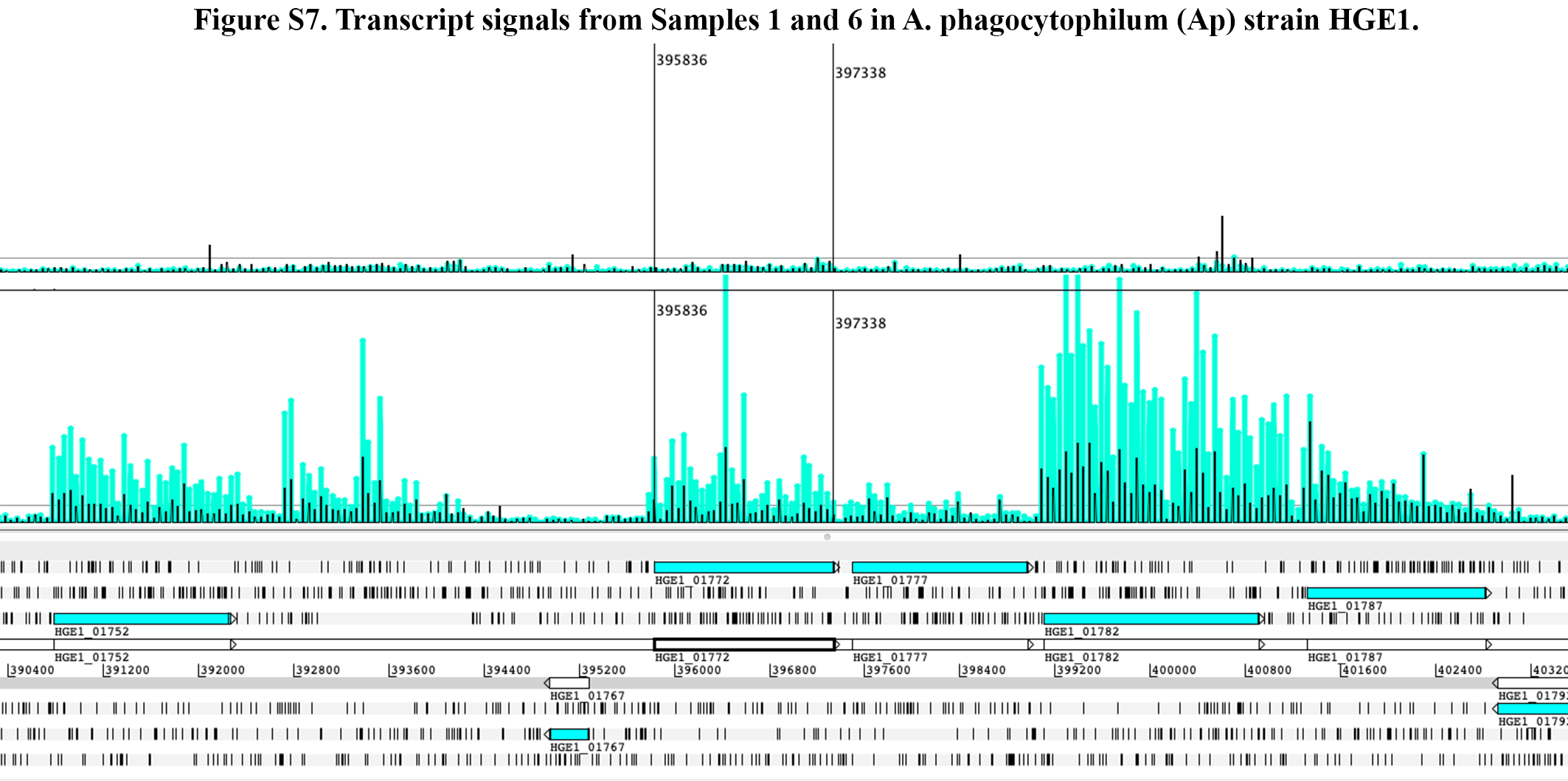

Supplement: Figure S7 — Transcription data (top two graphs) plotted over the annotated genome sequence (sections at the bottom) using the Artemis genome browser. Turquoise boxes denote coding regions of annotated genes. Each bar corresponds to one 60-mer probe on the tiling array. Green bars represent Ap transcription in sample 1 (2 h with HL-60), black bars represent Ap transcription in sample 6 (24 h in HL-60). Three annotated HGE-14 genes (HGE1_01752, HGE1_01772, and HGE1_01782) and one on the same DNA strand between HGE1_01752 and HGE1_01772 that is not annotated are upregulated at 2 h with HL-60. HGE-14 proteins are putative effectors predicted to enter the host nucleus and alter transcription (21, 22). Nucleotide positions in the genome are indicated by numbers above the gray line. [file Image_7.tif]

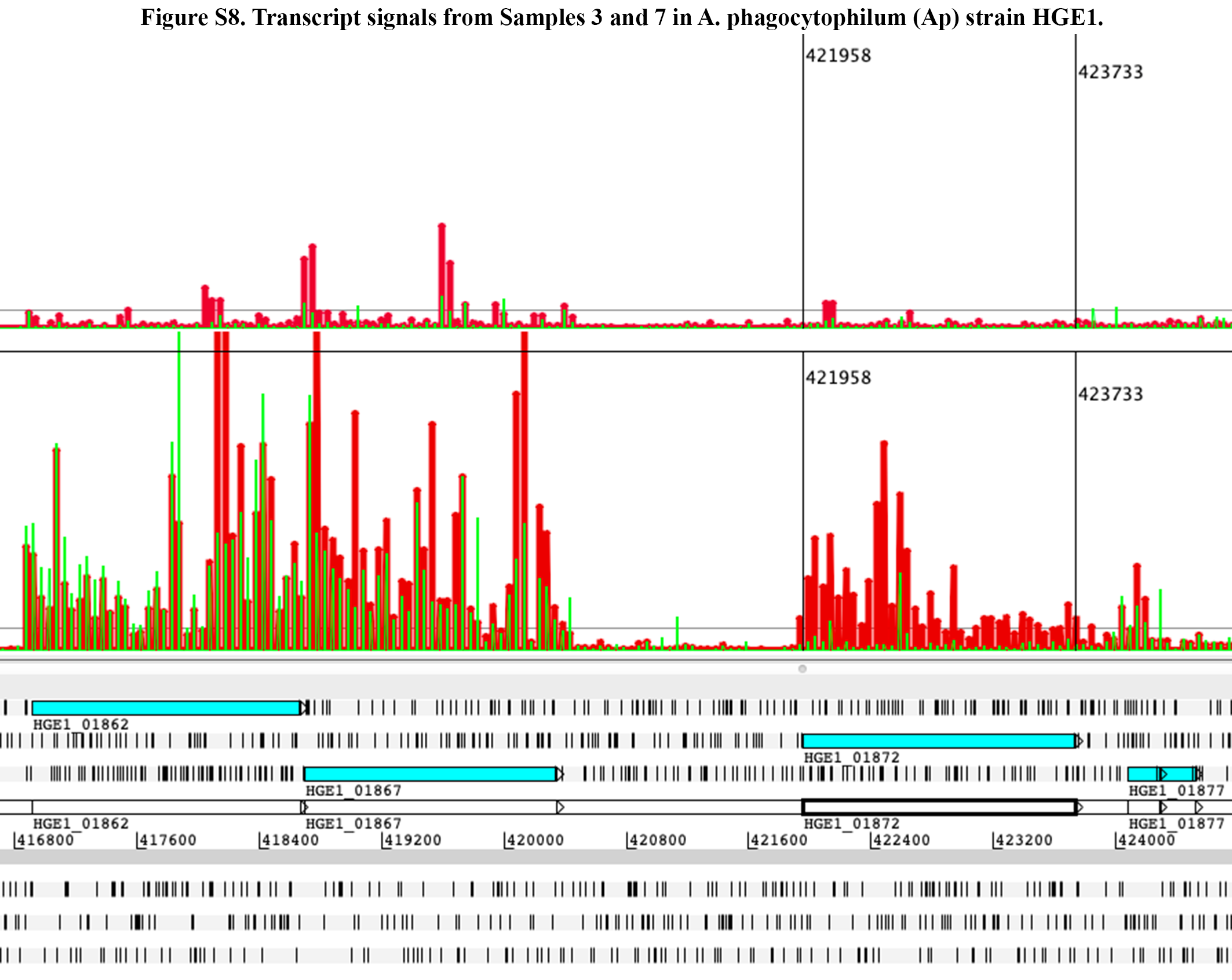

Supplement: Figure S8 — Transcription data (top two graphs) plotted over the annotated genome sequence (sections at the bottom) using the Artemis genome browser. Turquoise boxes denote coding regions of annotated genes. Each bar corresponds to one 60-mer probe on the tiling array. Green bars represent Ap transcription in sample 3 (2 h with ISE6), red bars represent Ap transcription in sample 7 (48 h in ISE6 cells). HGE1_01872, a hypothetical gene, is upregulated in replicating bacteria. Nucleotide positions in the genome are indicated by numbers above the gray line. [file Image_8.tif]

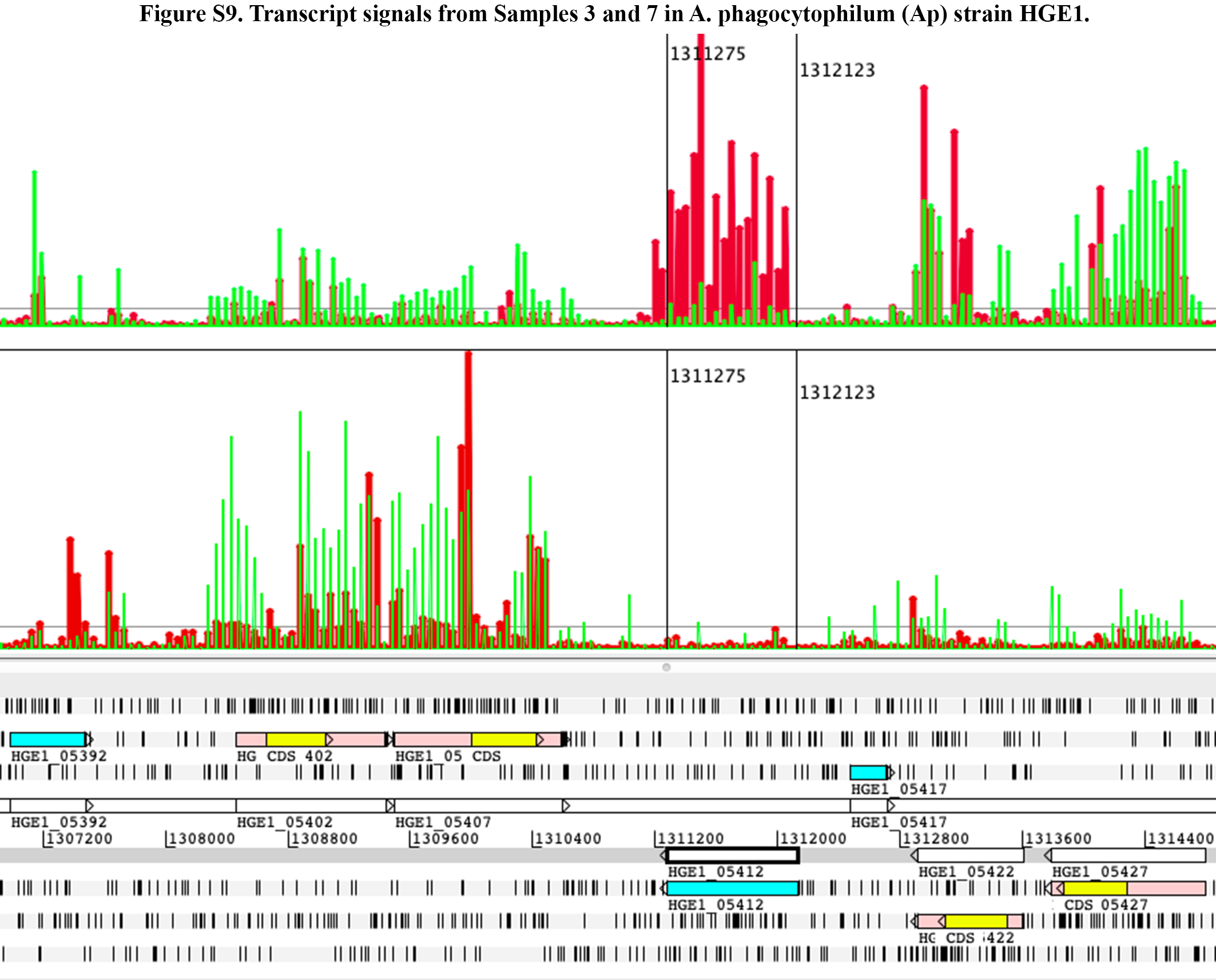

Supplement: Figure S9 — Transcription data (top two graphs) plotted over the annotated genome sequence (sections at the bottom) using the Artemis genome browser. Turquoise boxes denote coding regions of annotated genes, and pink boxes denote p44 conserved sequences with yellow boxes denoting their center hypervariable region. Each bar corresponds to one 60-mer probe on the tiling array. Green bars represent Ap transcription in sample 3 (2 h with ISE6), red bars represent Ap transcription in sample 7 (48 h in ISE6 cells). HGE1_05412 encoding MSP4 is upregulated during bacterial replication, whereas three p44 paralogs (HGE1_05402, HGE1_05407, and HGE1_05427) are upregulated at 2 h with ISE6 cells. [file Image_9.tif]
